# Process evaluations of health-promotion interventions in sports settings: a systematic review

**DOI:** 10.1093/heapro/daad114

**Published:** 2023-09-18

**Authors:** Angie S X Lim, Matthew J Schweickle, Caitlin Liddelow, Sarah K Liddle, Stewart A Vella

**Affiliations:** Global Alliance for Mental Health and Sport, School of Psychology, University of Wollongong, Northfields Avenue, Wollongong, New South Wales, 2522, Australia; Global Alliance for Mental Health and Sport, School of Psychology, University of Wollongong, Northfields Avenue, Wollongong, New South Wales, 2522, Australia; Global Alliance for Mental Health and Sport, School of Psychology, University of Wollongong, Northfields Avenue, Wollongong, New South Wales, 2522, Australia; Turner Institute for Brain and Mental Health, School of Psychological Sciences, Monash University, 18 Innovation Walk, Clayton, Victoria, 3800, Australia; Global Alliance for Mental Health and Sport, School of Psychology, University of Wollongong, Northfields Avenue, Wollongong, New South Wales, 2522, Australia

**Keywords:** implementation science, health-promotion, settings-based work, process evaluation, sports

## Abstract

Sports settings have been identified as an ideal place to conduct complex multi-level health-promotion interventions, with the potential to engage a broad audience. Whilst the benefits of delivering health-promotion interventions in sports settings are well documented, such interventions’ real-world implementation and success must be better understood. Process evaluations can be conducted to provide information related to an intervention’s fidelity, replication, scaling, adoption, and the underlying mechanisms driving outcomes. This systematic review summarizes how process evaluations of health-promotion interventions are conducted in sports settings and highlight facilitators and barriers to health-promotion intervention delivery using narrative synthesis. Following the Preferred Reporting Items for Systematic Reviews and Meta-Analysis guidelines, searches included original peer-reviewed articles from inception—January 2023. We searched eight electronic databases: Academic Search Complete; MEDLINE, PsycARTICLES; PsycINFO; SPORTSDiscus with Full Text; MEDLINE; SCOPUS; Pub Med, and Pro Quest Central. Thirty-two studies were included. Findings suggest that most process evaluations of health-promotion interventions have acknowledged the inherent complexity of sports settings, and investigated factors that explain their intervention’s success (e.g. trust building, engagement). However, poor use of process evaluation frameworks or guidelines resulted in wide variations of how process evaluations are conducted and reported, which made findings difficult to integrate and standardize with consistency. Accordingly, this review provides a guide on how future process evaluations can be conducted to improve health-promotion interventions’ transparency, replicability and reliability in real-world settings.

**Contribution to Health Promotion**

- This systematic review is the first to report on process evaluations of health-promotion interventions in sports settings.
- We highlight the core features that aid health-promotion intervention delivery in sports settings.
- Results indicate wide variations in how process evaluations of health-promotion intervention in sports settings have been conducted and reported.
- We provide recommendations to guide future health-promotion intervention development to ensure intervention impact, sustainability, engagement, feasibility and acceptability in sports settings.

## BACKGROUND

Globally, a large proportion of people regularly participate in sports, with approximately 80.5% of Australians above the age of 15 participating in a sport-related activity at least once per week (Clearinghouse for Sport, 2023). Further, approximately 44% of the European population and 63% of the United Kingdom population participate in sports at least once weekly (Eurostat, 2022; Statista Research Department, 2022). These high participation rates make sports settings (both at the professional and community level) an ideal place to engage with a broad audience for health-promotion initiatives, such as mental health-promotion, increasing physical activity, or nudge interventions involving health behaviours (Donaldson and Finch, 2012; Eime *et al.*, 2013; Kokko, 2014). Other research, however, has suggested that athlete populations had higher rates of both alcohol use and violence, higher incidences of sports-related injuries, and have traditional masculine norms and negative attitudes towards help-seeking, compared to non-athlete populations (Sønderlund *et al.*, 2014; Ramaeker and Petrie, 2019). Hence, whilst sports settings allow for population-wide engagement, considering sports clubs as health-promoting settings merely because of its sporting practices is reductive, due to its potential harmful effects on community health (Geidne *et al.*, 2019). Therefore, more research is needed to understand how the interplay of factors can enable and hinder (or constrain) implementation of health-promotion interventions within sports clubs.

Community sports (i.e. settings focused on fun and enjoyment whilst playing sport; Vella *et al.*, 2022) and professional sports (i.e. settings focused on competition where elite athletes often receive payment) have features that can facilitate effective health-promotion interventions. Sports settings have multiple layers of influence, including athletes, coaches, parents, club policies, national policies and legislation and cultural influences—all of which make health-promotion interventions difficult to implement (Kokko *et al.*, 2014). Though there is increased popularity in using settings-based health-promotion approaches in sports clubs, mechanisms underpinning the implementation and evaluation of their interventions need to be better defined and understood to improve such interventions’ real-world impact and efforts (Geidne *et al*., 2019; Van Hoye *et al*., 2021). These factors may include aspects related to fidelity, acceptability, replicability, maintenance of outcomes and translation of sports-based health interventions (Kokko, 2014; Vella *et al*., 2019; Hunt *et al*., 2020). To pinpoint how and why sports-based health interventions work in practice, it is necessary to consider how these factors interact to influence both intended and unintended intervention outcomes, using a complex systems perspective (McGregor, 2019).

Conducting process evaluations is one way to understand the contextual factors that may influence an intervention’s implementation process. Process evaluations are a type of program evaluation geared towards assessing the implementation process. Process evaluations are studies that run parallel to (or following) intervention trials, which attempt to conclude how well the project successfully followed the strategy laid out in the research plan (Byng *et al*., 2008; Grant *et al*., 2013). A robust process evaluation considers the intervention’s content, causal assumptions and mechanisms of change (Byng *et al*., 2008; Grant *et al*., 2013; Fridrich *et al*., 2015; Moore *et al*., 2015). Process evaluations are particularly effective in helping evaluators explain unexpected outcomes and understand the impact of context on outcomes (Robbins *et al*., 2011; Grant *et al*., 2013). Additionally, process evaluations assist in distinguishing between implementation failure (i.e. lack of expected results due to poor delivery practices) and theory failure (i.e. lack of results due to incorrect theory adoption; Stame, 2010).

A robust process evaluation aids in replicating, translating, scaling up and improving health-promotion interventions in real-world settings. The information from a process evaluation can allow sports organizations, charities and government bodies to more efficiently identify and allocate resources regarding health-promotion intervention scaling (Duflo, 2004). Further, clarifying real-world causal mechanisms can highlight how health-promotion interventions should be delivered to promote optimal outcomes at scale (Robbins *et al*., 2011; Grant *et al*., 2013; Moore *et al*., 2015). As such, conducting process evaluations can aid in explaining why and in what respect an intervention works; with what outcomes (and why); for whom and under what circumstances an intervention works; and whether sustained effects are observed (Pawson and Manzano-Santaella, 2012).

In this systematic review, we synthesized studies that had conducted a process evaluation of a health intervention delivered in all sports settings (i.e. professional and community sports). We aimed to summarize how process evaluations have been undertaken in a sports setting and highlight the key facilitators and barriers to health interventions. Although not our primary aim, we also identified process evaluation frameworks used to evaluate the implementation of interventions in sports settings. Ultimately, this review will inform future intervention development and planning, and guide how future process evaluations can be conducted to improve interventions’ transparency, replicability and reliability in sports settings.

## Methods

### Protocol and registration

This review was not pre-registered in PROSPERO as it did not contain ‘at least one outcome of direct patient or clinical relevance’ (Centre for Reviews and Dissemination, 2016). However, a search was conducted in PROSPERO for similar reviews to avoid duplication of the research. We adhered to the guidelines in the PRISMA Statement (Supplementary File 1; Page *et al*., 2021).

### Eligibility criteria

We established inclusion and exclusion criteria to ensure the scope of the review was clearly defined and that all literature relevant to the review’s aims was identified. We included interventions that regarded sports clubs as a vehicle for conducting health-promoting interventions due to the wide reach of sports settings. We also included interventions that aim to develop health-promoting activities within a sports system, whereby the health-promoting actions support the core business (i.e. sports performance, health and development) of club members (Whitelaw, 2001; Kokko, 2014; Geidne *et al*., 2019; Van Hoye *et al*., 2021).

Articles were included if: (i) the study explicitly stated that they conducted a process evaluation of any health interventions targeting any age groups, genders and intervention focus (i.e. interventions that had a sports component, but also another aspect of health, such as diet and mental health interventions); (ii) constituted original empirical research; (iii) was a peer-reviewed journal article published in English; (iv) the context of the intervention meets the definition of ‘sport’ (i.e. community sports clubs, professional sports clubs). We defined sport in this review as human activities involving physical exertion and skill as the primary focus of the activity, with elements of competition or social participation that take place in an organized setting, following a set of rules (i.e. football/soccer, rugby; Clearinghouse for Sport, 2023). We excluded articles if: (i) studies did not explicitly use the term ‘process evaluation’; (ii) any process evaluations of interventions not performed in a sports setting (i.e. schools, colleges, universities, or workplaces); (iii) not empirical research.

### Information sources

#### Search strategy and electronic sources

Following initial scoping searches by the first author on the EBSCOhost database, we developed a comprehensive search strategy through critical discussions between all authors. The search string employed the following Boolean search terms:

sport* OR athlet*

AND interven* OR workshop* OR program

AND (‘process evaluation’)

We adapted the search string for each database as required. Results were limited to peer-reviewed journals in the English language. All blocks in the search string were searched at the full-text level to maximize search results. Truncation symbols were applied, where possible, to ensure we captured the breadth of a term’s variant spellings (Supplementary File 2).

Searches were conducted in June 2021 (inception—2021) and then updated in March 2022 (2021–2022) and January 2023 (2022–2023). We searched eight electronic databases: Academic Search Complete; MEDLINE, PsycARTICLES; PsycINFO; SPORTSDiscus with Full Text; MEDLINE; SCOPUS; Pub Med and Pro Quest Central. The first author conducted a further manual literature search to ensure that all relevant studies were included. Reference lists of all identified studies were searched, in addition to forward searching citations using Google Scholar. The first author repeated the manual literature search process with each new study added.

### Screening

#### Data management and selection process

References identified were imported and screened in Covidence (www.covidence.org, 23 December 2022), a web-based systematic review management tool. In Covidence, two screeners (A.L. and S.L.) independently screened articles at the title, abstract and keyword levels for relevance. We obtained the full-text article of the remaining studies. Two reviewers (A.L. and C.L.) assessed these studies independently against the eligibility criteria. At least two reviewers (A.L. and C.L.) from the authorship team undertook the same process for updated searches and further identified studies. Disagreements were resolved through critical discussions among the reviewers or, if necessary, discussions with a third and fourth reviewer from the authorship team (M.S. and S.V.).

#### Data extraction and narrative synthesis

We followed Popay *et al.*’s (2006) guidance on systematic reviews for implementation studies to guide data extraction. The first author independently extracted relevant process evaluation data from included studies. M.S. and C.L. checked a random sample of the data extracted (*n* = 15%) from the studies to ensure the integrity of the findings. Data extracted included: intervention description, process evaluation study design, process evaluation participant characteristics, process evaluation frameworks and process evaluation outcomes tested. We explored and integrated key findings from the included studies through narrative synthesis (Popay *et al*., 2006). Following the narrative synthesis process, we textually described the overall findings while noting key differences in study characteristics, and implementation, to generate new insights (Popay *et al*., 2006).

### Quality appraisal

As no quality appraisal of process evaluations exists, we created a quality appraisal checklist informed by Grant *et al*. (2013) to operationalize the proposed framework for conducting process evaluations (Supplementary File 3). This was achieved by extracting the core methodological features in designing and reporting process evaluations, highlighted in Grant *et al*. (2013)’s findings. Subsequently, we constructed a checklist based on the core methodological features identified—comprising 14 items (including sub-items)—assessing the quality of the process evaluation undertaken. Half-points were given to items that had two parts to a criterion. For example, ‘Process evaluation clearly reported if they were: (i) pre-specified or post hoc (0.5), and (ii) why the selected timing was chosen (0.5)’. Of a total possible score of 14 points, we scored each item on a point basis (i.e. yes= 1 or 0.5; no= 0) to yield a summary score for each study. AL reported the summary score of each study included in the review (Supplementary File 4). To ensure scoring was conducted appropriately, C.L. and M.S. appraised a random sample of 20% of the included studies. There were no discrepancies between the scores, and subsequently, the quality appraisal was deemed reliable.

## RESULTS

### Search returns

**Fig. 1:**
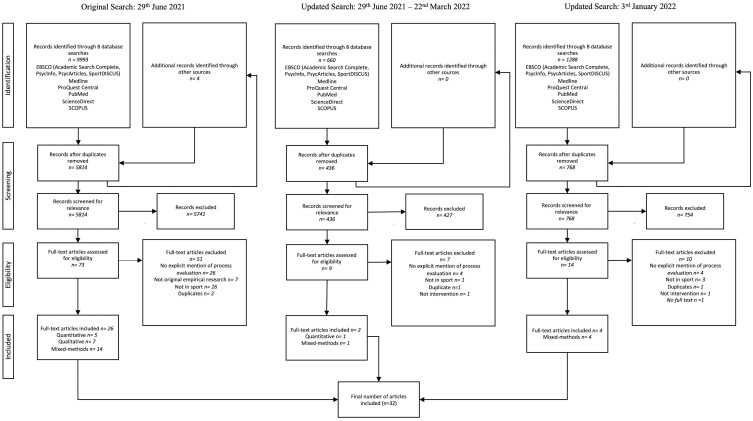
The PRISMA flow chart.

We have presented the search results in the PRISMA flow diagram (Figure 1). After removing duplicates, two reviewers (A.L. and S.L.) independently screened 6249 articles by title and abstract in duplicate. A total of 6157 records were excluded for failing to meet the predefined eligibility criteria at this stage. The remaining 73 articles were screened at the full-text level, of which 26 studies were included in the review. The first author conducted second and third searches in June 2022 and January 2023 using the same search criteria. However, the date range was restricted to only include studies published after the previous search date (i.e. second search: June 2021, third search: January 2023). Six additional studies were identified, resulting in 32 articles (Figure 1).

### Quality assessment

Scores ranged from 6.5 to 13 points (Supplementary File 4). The quality appraisal checklist yielded an average study quality score of 10.8 across all studies, out of a possible 14 points. All studies included tailored the process evaluations to the trial, clearly specified that a process evaluation was undertaken, and clearly stated the purpose of the evaluation. However, 47% (*n*=15) of studies did not use a process evaluation framework, and only 11 studies (34%) explicitly investigated the unintended outcomes of the intervention. Whilst all studies considered the effects of the intervention’s primary outcomes in their process evaluation, only seven studies (21%) considered secondary outcomes.

### Summary of included studies

Supplementary File 5 describes the process evaluation aims of all included studies, including the purpose, research questions and process evaluation measures. We extracted: the aims of their process evaluation; sample size and participant characteristics of the process evaluation; intervention study design; intervention descriptions; process evaluation data collection and follow-up time points; process evaluation methods; process evaluation frameworks; and outcome measures used. All process evaluation studies in this review conducted health-promotion interventions, including mental health literacy and youth development interventions, physical activity and weight-loss interventions, interventions encouraging healthier eating behaviours and male health-promotion and recovery interventions.

### Characteristics of process evaluations

We report details containing the characteristics of process evaluations in this study in online Supplementary File 5. Nine studies reporting from the same interventions adapted across different regions (Football fans in training adaptations, *n=*6; ahead of the game, *n=3*). Most of the process evaluation studies (65.6%; *n*=21) were mixed methods, four were quantitative, and seven were qualitative. A majority (*n*=16) only looked at the participants’ perspectives and experiences of participating in the intervention. Twelve studies conducted process evaluations using a mixed sample (i.e., the perspective of facilitators and participants), and three studies considered only the facilitator’s perspective in their process evaluation.

Of the 32 studies, 17 reported using a framework, recommendation, or guideline for their process evaluation. Of the 17 studies that reported using a framework, the most common framework was the Medical Research Council framework (*n* = 5). Four studies combined several different frameworks in their process evaluation. For example, Vella *et al*. (2019) combined the National Implementation Research Network (Fixsen *et al*., 2005), Saunders *et al*. (2005) and the Consolidated Framework for Implementation Research (Damschroder *et al*., 2009) in their evaluation. In addition, several other frameworks and recommendations for designing process evaluations were identified during data extraction. These frameworks included: PRACTIS guide (Koorts *et al*., 2018); Nielsen *et al*. (2010); Bowen *et al*. (2009); Burnette and Mazzeo (2020); Issel (2004); Proctor *et al.* (2011) framework of implementation outcomes; and Roger’s Diffusion of Innovation theory (Rogers, 2003).

### Indicators of successful implementation in process evaluations

We identified several indicators for successful implementation by extracting data specific to how the included studies defined, observed and determined the successful (or otherwise) implementation of their intervention (Supplementary File 6). Specifically, these included intervention engagement (i.e. participants uptake and adherence; *n* = 16), intervention completion (i.e. extent to which participants completed the intervention components; *n* = 8), intervention delivery (*n =* 15), intervention feasibility (*n =* 11), participants’ perceptions of the program *(n=* 12), whether the intervention had a positive impact on intended program outcomes (*n=* 9), and how well the intervention reached the target population (*n=*7). However, fewer studies explicitly reported differences between the expected and actual delivery of their program (*n=*17) – all of which are helpful in assessing the successful implementation of an intervention (Supplementary File 6).

### Facilitators

Several factors were identified as facilitators and barriers to health-promotion intervention delivery across the studies included in this review (Table 1).

**Table 1:** Facilitators and barriers to intervention delivery identified through process evaluations

	Themes	Key content	References
*Facilitators*	**Facilitators of intervention delivery**		
	Setting and context of intervention	Association with premier league football club setting due to convenience, congruence with masculine identities, safe and familiar social space, provided insider view of club.	Gray *et al*. (2013), Hunt *et al*. (2014, 2020), Kwasnicka *et al*. (2022), Maddison *et al*. (2019), Parnell *et al*. (2015), Robertson *et al*. (2013)
	Resources and logistics are well placed within the club	Having the appropriate facilities, club infrastructure, training and equipment to support the delivery of interventions.	Brooke *et al*. (2022), Hägglund *et al*. (2021); Hunt *et al*. (2020), Kwasnicka *et al*. (2022), Naylor *et al*. (2015), Newman *et al*. (2021), O’Brien *et al*. (2021), Petrella *et al*. (2022), Robertson *et al*. (2013); Sandgren *et al*. (2022), and Vella *et al*. (2019)
	**Facilitators of participation and engagement**		
	Building trust	Trust in the credibility of an intervention encouraged intervention reach, uptake and continuation.	DeCelles *et al*. (2016), O’Brien *et al*. (2021), Robertson *et al*. (2013)
	Intervention/program relevance to the context	Program that are straightforward to implement, tailored and designed to address the needs of the participants, interactive and practical program components improved engagement.	Chen (2020), Hurley *et al*. (2018). O’Brien *et al*. (2021), Petrella *et al*. (2022), Sandgren *et al*. (2022), Wynters *et al*. (2021); Brooke *et al*. (2022) and Hägglund *et al*. (2021)
	Encourage belongingness	Interventions encouraging belongingness with continued participation motivated participants to continue with the intervention.	Gray *et al*. (2013), Hunt *et al*. (2014), Fuller *et al*. (2014), Kwasnicka *et al*. (2022), Brooke *et al*. (2022), Parnell *et al*. (2015), Petrella *et al*. (2022), Lauwerier *et al*. (2020), Dunn and Holt (2004)
*Barriers*	**Barriers to intervention delivery**		
	Lack of resources due to logistic challenges	Difficulties sourcing products due to specific characteristics (i.e., low shelf life of healthier products), concerns regarding budget constraints and revenue loss, and lack of available infrastructure and internal resources within community club setting to support intervention delivery.	Naylor *et al*., (2015). Roncarolo *et al*. (2015), Van Rookhuijzen and De Vet (2021) and Vella *et al*. (2019)
	Lack of buy-in from stakeholders	Lack of alignment between clubs’ interest and intervention vision.	Naylor *et al*. (2015), Van Rookhuijzen and De Vet (2021)
	Delivery time and constraints	Time constraints and difficulties scheduling workshops.	Brooke *et al*. (2022) Chen (2020), Lauwerier *et al*. (2020), Maddison *et al*. (2019), Panza *et al*. (2022). Petrella *et al*. (2022), Wynters *et al*. (2021)
	**Barriers to participation and engagement**		
	Competing demands	Competing responsibilities of participants, stakeholders and delivery staff led to difficulties balancing multiple priorities along with the intervention requirements.	Chen (2020), Gray *et al.*, 2013, Lauwerier *et al*. (2020), Maddison *et al*. (2019), McGregor (2019), Naylor *et al*. (2015), O’Brien *et al*. (2021), Panza *et al*. (2022), Petrella *et al*. (2022), Roncarolo *et al*. (2015), Van Rookhuijzen and De Vet (2021) and Vella *et al*. (2019)
	Motivation	Difficulties creating and improving participants’ perceived value towards intervention.	Brooke *et al*. (2022), Hunt *et al*. (2020), Hurley *et al*. (2018), Petrella *et al*. (2022)

#### Facilitators of intervention delivery

##### Setting and context of intervention

The premier league sport setting and team environment were strong facilitators for intervention uptake, and completion (*n=* 7). These included: the engaging setting of sports clubs, the propensity for sports clubs to provide a safe, familiar and non-stigmatizing social space especially for mental health interventions, the prestige of the football club setting, the convenience of accessing sports clubs, and sports clubs being a location that is congruent with masculine identities, particularly for interventions developed for males. For example, ensuring that locations are congruent with masculine identities, and were in convenient locations, such as Premier League sports clubs, was helpful in attracting participants for interventions designed for men. Additionally, the football club setting promoted a safe and familiar social space that generated trusting engagement among participants, contributing to their intervention success (Robertson *et al*., 2013). The Premier League football club setting also provided an insider view into the club, which incentivized participant engagement and intervention uptake, as participants had the opportunity to feel more connected with the professional football club (Parnell *et al*., 2015). Together, these studies highlighted the importance of considering the context in which the intervention occurs, and how the sports club setting can facilitate intervention success.

##### Resources and logistics are well-placed within the club

Clubs with resources to support intervention delivery helped facilitate intervention success (*n=*11). These resources included: facilities to support indoor implementation, the capacity to provide training and technical support, clubs with the infrastructure to support collaboration with intervention deliverers, and the appropriate facilities and equipment to support the delivery of interventions. Clubs having the appropriate facilities and equipment, such as indoor locations to support the delivery of interventions, facilitates intervention success, as it reduces levels of distraction and allows participants to better engage with materials. Therefore, having the capacity and resources to provide training and technical support is a meaningful consideration for intervention delivery.

#### Facilitators of participation and engagement

##### Building trust

Trust in the credibility of interventions, through community connections and stakeholder buy-in (i.e. coaches, community services), was helpful in facilitating intervention uptake, participation and continued engagement with health-promotion interventions in sports settings (*n =* 3). Trust was facilitated through community connections, word of mouth and the importance of trusted relationships between friends and members of a community (Robertson *et al*., 2013). Particularly, advertising interventions through established and trusted community partnerships, such as the UK National Health Services, helped increase their intervention uptake due to participants’ increased trust in their program’s credibility (Robertson *et al*., 2013). In addition, buy-in from the head coach also improved intervention credibility, resulting in improved intervention uptake (DeCelles *et al*., 2016; O’Brien *et al*., 2021). The community buy-in and endorsement of interventions, therefore, assist in encouraging intervention participation, uptake and continuation, given the established trust with participants.

##### Program relevance to the context

Programs tailored and designed to address the specific needs of the participants were strong facilitators for implementation (*n =* 8). Specifically, personalizing the intervention components to meet the individual needs of the participants was valuable for implementation success. Indeed, participants who felt their personal goals needed to be more relevant to the intervention component were also less motivated to complete their assigned tasks or partake in the intervention. Therefore, content that explicitly addresses participants’ needs, aims and motivations is helpful for improving participants’ engagement with and receptiveness towards an intervention.

##### Encourage belongingness

Interventions that have the propensity to encourage belongingness with continued participation helped improve implementation (*n* = 9). Having coaches and intervention facilitators whom participants found relatable (i.e. with similar shared experiences) encouraged trust and camaraderie within the group. Participants in group-based interventions reported that while the sports setting attracted them to the intervention, the belongingness fostered in the intervention encouraged continued engagement with the program. Thus, while interventions that encourage trust are important for engagement, consideration for the role of belongingness is also helpful in encouraging continued engagement with an intervention.

### Barriers

#### Barriers to intervention delivery

##### Lack of resources due to logistic challenges

The need for more resources due to logistic challenges affected implementation (*n* = 4). These resources included: having a consistent, enclosed training to deliver the workshops; program budget constraints; scarce community club resources; and lack of intervention delivery personnel. Community sports clubs are often under-resourced and underfunded, with most volunteers holding multiple responsibilities within the clubs (i.e. being both president and coach; Finch and Donaldson, 2010). Given the core business of sports clubs is to promote participation in sport and physical activity; consequently, having the resources to support intervention delivery and evaluation within the club is challenging—especially among coaches and other delivery personnel who hold multiple club responsibilities are typically more prepared, trained and committed to practical activity delivery. Therefore, ensuring the goals of the intervention are sustainable by considering a club’s available infrastructure and internal resources may aid in improving implementation fidelity.

##### Lack of buy-in from stakeholders

As health-promotion initiatives within the sports setting typically take a multi-level, ecological approach (Kokko *et al*., 2014), requiring more alignment with the clubs’ interests and the intervention vision impacted implementation (*n* = 3). For example, in non-core business interventions aiming to promote healthier dietary choices across sports clubs, stakeholders felt that the responsibility to promote a healthy diet was not their responsibility but that of parents or guardians. Further, healthier choices being uncommon in football clubs raised concerns about lower revenue for the club from fans purchasing food, impacting the acceptability of the intervention. Finally, as the intervention was not the sports clubs’ priority, club personnel found it challenging to implement the program according to plan during peak-meal hours whilst serving multiple customers. Therefore, the stakeholders’ interests in delivering and supporting interventions within the club setting must be well-aligned with the intervention goals to ensure smoother delivery.

##### Delivery time and constraints

Difficulties in finding a suitable time to schedule workshops and having sufficient time to deliver all workshop components hindered implementation (*n*= 7). In addition, due to time constraints, some program deliverers only delivered the core contents of their intervention, with activities and exercises omitted to a minor extent (Lauwerier *et al*., 2020; Maddison *et al*., 2019). Therefore, whilst adhering to program content is important, ensuring that interventions are delivered succinctly should be considered for delivering interventions in sports settings.

#### Barriers to participation and engagement

##### Competing demands

Competing demands of participants and delivery personnel impacted program engagement and adherence (*n=*12). These included: participants’ and intervention staff’s work commitments, family commitments, health barriers, personal and psychosocial issues and interference with the athletes’ training schedule. Notably, coaches were unwilling to sacrifice training time for the intervention (Panza *et al*., 2022), and some participants were unavailable before or after training to participate in the interventions (Vella *et al*., 2019). Additionally, interventions delivered by coaches were impacted by difficulties balancing their many responsibilities within the club (e.g. teaching skills, planning competitions, organizing events and managerial responsibilities). Delivery personnel in clubs that function on a non-core business model had concerns regarding product sales, and had difficulties adhering to nudge interventions promoting healthier food options during peak business hours (Naylor *et al*., 2015, Van Rookhuijzen and De Vet, 2021). Therefore, these competing demands impacted intervention reach as delivery personnel were unable to follow the intervention goals whilst managing multiple responsibilities, which impacted participants engagement with the program.

##### Motivation

A few aspects impacted participants’ motivation to engage in the intervention, influencing implementation (*n=* 4). These included: stigmatizing attitudes towards culturally sensitive topics, increasing participants value towards a program, and encouraging continued participation in the program. An intervention targeting weight loss for men found that addressing sensitive topics challenging certain cultural norms influenced participants’ motivation to participate in an intervention (Hunt *et al*., 2020). A mental health literacy intervention for parents of adolescent males found that increasing participants’ perceived value towards the program was challenging, which limited intervention reach and engagement (Hurley *et al*., 2018). Thus, creating perceived value towards an intervention whilst carefully considering the needs, cultural attitudes and considerations of the intervention topic is helpful in improving participants’ motivation to engage with an intervention.

## Discussion

### Summary of findings

This review aimed to summarize how process evaluations have been conducted in sports settings and highlight facilitators and barriers to interventions in sports settings. We synthesized data from 32 studies to address these aims. Specifically, we synthesized the indicators used to measure successful implementation, along with common facilitators and barriers reported across studies. Although not the primary aim, this review also considered the process evaluation frameworks used to evaluate the implementation of health-promotion interventions in sports settings. Overall, we found that process evaluations of health-promoting interventions in sports settings lacked consistency, transparency and clear reporting of data. Few studies adopted a multi-method, multi-perspective approach and only included either the participants’ or deliverer’s perspectives in their process evaluation. We also identified that facilitators and barriers to intervention engagement and delivery are often viewed dichotomously, without consideration that some facilitators may also act as barriers. Subsequently, we identified several core features worth considering for future intervention and process evaluation designs.

## RECOMMENDATIONS FOR CONDUCTING PROCESS EVALUATIONS IN SPORTS

### Low quality of process evaluations in health-promotion interventions in sports settings

More consistency and transparency in how process evaluations are conducted are needed, and process evaluations should be underpinned by a theoretical evaluation framework (Grant *et al*., 2013; Moore *et al*., 2015; Glasgow *et al*., 2019; Pawson, 2019). Frameworks guide the consideration of factors important for process evaluations, including contextual factors, causal assumptions and expected or unexpected factors influencing outcomes. Whilst the studies included in this review shared commonalities in how implementation success was observed in their interventions (i.e. engagement, participation and adherence), nevertheless, most process evaluations in this review did not adhere to any process evaluation framework; the studies that did largely varied in the frameworks used. Hence, the wide variance in how process evaluations are conducted warrants more clarity in reporting, which will improve replication, integration and implications (McGill *et al*., 2021). Accordingly, targeted frameworks suitable for assessing complex interventions (i.e. Medical Research Council Framework, RE-AIM, underpinned by realist evaluation methods) can improve the standardization in methodology and consistency in reporting data (Glasgow *et al*., 2019; Pawson, 2019). Future process evaluations should therefore utilize suitable evaluation frameworks to ensure that findings can be standardized and integrated consistently.

As a starting point for reporting process evaluations in sports settings, we recommend the following. The setting in which an intervention occurs (i.e. context) must be clearly labelled, as it is critical in evaluating and understanding the implementation of an intervention. Further, key features of the context (i.e. club structure, reach, demographic information) must also be clearly defined, most notably including the characteristics of participants observed within the process evaluations. In this study, there were inconsistencies in how demographic data were reported across included studies (i.e. missing data on age, gender and sample size). The lack of clarity regarding such key contextual information made it challenging to determine who is being investigated, the implication and generalizability of the findings, and how the context may have influenced the results. In future, researchers should ensure that process evaluations include accurate reporting of demographic data and provide more clarity on the assumptions underlying the context in which the intervention occurs.

## INCLUDING MULTI-METHOD, MULTI-PERSPECTIVE PROCESS EVALUATIONS OF HEALTH-PROMOTION INTERVENTIONS

Including multiple diverse perspectives is important in assessing a program’s implementation success and how sports-based intervention works in practice. For example, a robust observation—triangulating participant and facilitator data—results in a more thorough process evaluation. However, over two-thirds of the studies only considered a singular perspective in their process evaluation (*n =* 17). Whilst participants’ favourable perception of a program is valuable and provides insight into how well the intervention was received, more is needed to determine an intervention’s success and implementation process. For example, delivery staff perspectives can shed light on the unintended outcomes which arose during implementation and other stages, including evaluation. These perspectives obtained through diverse methods (i.e. fidelity sheets, recruitment logs and qualitative interviews) can provide input on the recruitment procedure and strategies, the feasibility of implementation as intended, as well as future considerations to help improve the implementation process. As these outcomes commonly arise from the social relationships to which participants belong, a holistic consideration of perspectives (i.e. participants, delivery staff and key stakeholders) is therefore important in understanding the mechanisms that drive intervention outcomes.

Different factors in sports settings can interact to produce desired (or undesired) effects, influencing the outcome of an intervention (Dooris, 2006). Hence, individuals are not independent of the social units to which they belong (Kokko *et al*., 2014). As one way of improving the conduct of process evaluations, researchers should consider developing multi-method process evaluations and interventions underpinned by theories that can account for such complexity, such as complex systems theory. Complex systems science, critical realism and realist evaluation principles integrated within multi-method process evaluations can build a more comprehensive understanding of causality within complex environments (Fletcher *et al*., 2016). A systems approach offers guidance to evaluators of empirical research. Specifically, it can inform process evaluation designs for applied interventions by directing evaluators to consider simultaneous external factors which may influence outcomes. Whilst not specifically intervention-focused, such perspectives can provide valuable insight into the complex nature of multi-level health interventions which are commonplace in sports (McGill *et al*., 2021). Accordingly, suitable frameworks underpinned by a systems approach can help researchers and practitioners who design and implement interventions consider all aspects that influence their evaluation. Future process evaluations of interventions in sports should thus reflect this complexity by including multi-perspective and multi-method approaches (i.e. including both participation and delivery perspectives; supplementing qualitative methods with quantitative data), and adopt process evaluation frameworks that consider contextual factors to guide evaluations.

## DICHOTOMOUS VIEW OF FACILITATORS AND BARRIERS IN PROCESS EVALUATIONS

It is important that facilitators and barriers are not viewed dichotomously (i.e. as mutually exclusive) across the included studies. However, it is important to acknowledge that whilst certain factors can help facilitate intervention success, they may also act as barriers to implementation. For example, the findings demonstrated that although delivering sports-based interventions in a team setting helps encourage trust among participants, having too many participants may lead to higher levels of distraction, which can negatively impact participant engagement (Wynters *et al*., 2021; Brooke *et al*., 2022). Future intervention planning, therefore, should also consider how and under what circumstances identified facilitators might act as barriers to maximize implementation success. This requires balancing the benefits and detriments of conducting interventions in the sport setting to optimize intervention fidelity and effectiveness.

## RECOMMENDATIONS FOR CONDUCTING INTERVENTIONS IN SPORTS SETTINGS

Drawing upon the findings of this review, we provide a few recommendations for conducting health-promotion interventions in sports settings. Firstly, fostering belongingness and trust provided participants with engagement away from the intervention by encouraging social support amongst one another, which contributed to success across several interventions. Considering group facilitation strategies to improve the interpersonal dynamics between participants—such as incorporating ice-breaking activities at the beginning of the workshop—could help improve engagement and continuation of the intervention. Second, as concerns regarding scarce club resources that impact an intervention’s feasibility arose, implementers can mitigate such barriers by providing necessary resources (i.e., funding, training, delivery personnel) to lessen the burden of participation and intervention delivery. Third, the included studies reported difficulties scheduling the intervention due to competing time demands and club priorities. Implementers can address these barriers by adopting flexibility in their approach to scheduling workshops—such as preparing options to reschedule workshops—to improve the intervention’s acceptability, fidelity, engagement, reach and program completion. Finally, it is also worth considering the complexity of factors and the multiple levels of influence that contribute to implementing interventions in the unique context of sports. Thus, implementers can capitalize on strategies to improve stakeholder buy-in to ensure the intervention can be delivered to the target population. Future health-promotion intervention designs using sports settings as a vehicle of delivery, therefore, should consider the nuances involved in the implementation and communication of their intervention to mitigate the challenges that present with complex interventions.

## STRENGTHS, LIMITATIONS AND FUTURE DIRECTIONS

This review is the first to synthesize data from process evaluations of interventions in sports settings. Adhering to the process evaluation guidance proposed by Grant *et al*. (2013) assisted in organizing findings and enabled a robust synthesis of the included studies. This process also enabled the development of textual narrative summaries detailing how process evaluations have been conducted in sports settings and provided essential aspects to consider for future intervention development in sports settings. However, given the lack of consistency in how process evaluations are conducted, this review only included studies that have explicitly mentioned conducting a ‘process evaluation’ in the full text. As such, studies that may constitute a process evaluation, but have not named it as such, were not included in this review. Similarly, a study may claim to have conducted a process evaluation, but there is no guarantee of its rigour or quality. Further, ambiguities surrounding the context in which the intervention took place led to the exclusion of several studies, and are symptomatic of the poor reporting of contextual information. While this allows for standardization and better integration of findings, some studies may have been missed or excluded for this reason. Furthermore, as no current quality appraisal for process evaluations exist, this study developed a quality appraisal checklist for the studies included. Whilst this provides a template for future research to consider how to conduct process evaluations and assist in the standardization of the quality of process evaluations, the validity and internal consistency of the measure were not assessed. As such, future research can consider developing a validated measure to assess the quality of process evaluation, which encourages standardization of measures and quality appraisal of future process evaluations.

## CONCLUSION

This systematic review synthesized how process evaluations of sports-based interventions have been conducted and highlighted the core features that aid the delivery of health-promotion interventions in sports settings. Whilst most process evaluations acknowledged the complexity inherent in sports settings and investigated factors that explain their intervention’s success, these were limited by a lack of framework or guidelines. Subsequently, there was wide variation in how the process evaluations were conducted. However, the results highlighted the core features that contributed to intervention delivery success. For example, interventions that: included activities to foster belongingness and trust among participants and program facilitators; provided clubs with the necessary resources to support intervention delivery; included strategies to improve stakeholder buy-in; ensured flexibility in scheduling workshops; and provided careful consideration toward group formation and dynamics—especially when an intervention involved stigmatized topics—greatly contributed to intervention success.

In future interventions and process evaluations, researchers should consider how the different levels of influence interact to produce desired (or undesired) outcomes by adopting a complex systems approach. The lack of guidance resulting from the poor use of frameworks makes it difficult for findings to be standardized or integrated with consistency, and crucial aspects to consider in process evaluations may have been missed. Therefore, suitable frameworks (i.e. MRC Framework; Moore *et al*., 2015), RE-AIM; Glasgow *et al*., 2019—or guidelines such as Saunders *et al*. (2005) and Grant *et al*. (2013)—combined with a system thinking approach, are required to refine and standardize how future process evaluations are conducted and reported.

## Supplementary Material

daad114_suppl_Supplementary_Material

## Data Availability

All data generated or analyzed during this study are included in this published article [and its supplementary information files].
