# Correction to: Process evaluations of health-promotion interventions in sports settings: a systematic review

**DOI:** 10.1093/heapro/daae026

**Published:** 2024-04-11

This is a correction to: Angie S X Lim, Matthew J Schweickle, Caitlin Liddelow, Sarah K Liddle, Stewart A Vella, Process evaluations of health-promotion interventions in sports settings: a systematic review, *Health Promotion International*, Volume 38, Issue 5, October 2023, daad114, https://doi.org/10.1093/heapro/daad114

The following changes were made to the original publication of this manuscript. In **Table 1**, in the column ‘**References’**, for the line ‘**Intervention/program relevance to the context’**, the last two references should read: “Brooke *et al*. (2022) and Hägglund *et al.* (2021)” instead of: “Brooke *et al*. (2022) and Petrella *et al*. (2022)”.

In the **Supplementary Data** material, in Electronic Supplementary File 5: p.14, line for ‘Hägglund et al. (2021) Sweden’, fourth column should read: “Age: M age = 51 years (SD 0.8) N = 18 (Phase 1 & 2: [n= 9]; Phase 3: [n= 9]) Gender: Males (63.63%)Sport: Phase 1 & 2: Sport of athletics; Phase 3: aesthetic sport, figure skating” instead of: “Age: Range (31-62) N = 17Gender: Males (38.8%)Sport: Phase 1 & 2 not available; Phase 3: aesthetic sport, figure skating”. p.28, line for ‘Hägglund et al. (2021)’, the second column should read: “Mindfulness self-reflection intervention (delivered via SMS) to support sustainable high-performance coaching.

Pilot: Daily SMS intervention conducted with 11 national coaches, over 5 consecutive competition days, during the 2016 European Championship.

Phase 1: SMS-diary trialed daily for 8 consecutive weeks (N=9). Prior to beginning the intervention, a lecture was delivered to introduce the principles of mindful self-reflection and self-awareness and explain the associated benefits to help support a sustainable HPC career. Coaches also received a 90-second video clip explaining components of the daily SMS-intervention (i.e., ratings of energy and mood, and how to report their highlight of the day).

Phase 2: 1 month after the completion of the daily SMS-diary (Phase 1), a weekly intervention began with the same 9 participants. Participants received the SMS at 9 p.m. each Sunday and on each Monday an SMS reply to summarize participant’s reflections on the preceding 7 days.

Phase 3: Nine figure skating high performance coaches engaged in the SMS-dairy intervention for 8 weeks following a lecture on mindful self-reflection, self-awareness, and the role they have in facilitating sustainable high performance coaching career. This intervention was intentionally undertaken during the most demanding coaching period in the 4-year Olympic cycle, co-occurring with the National Championship and the qualification period for the Olympic Games. The fidelity and reach of the intervention remained high despite the context in which the intervention was delivered.”

instead of:

“Mindfulness self-reflection intervention (delivered via SMS) to support sustainable high-performance coaching.” And the fourth column should read:

“Mindfulness interventions among coaches will help support and impact sustainability in the high-performance coach profession. Coaches who can be present and aware are able to better assist athletes and prevent burn out.” instead of:

“Mindfulness interventions among coaches - coaches who are able to be present and aware are able to better assist athletes and prevent burn out”.

p.41, line for ‘Hägglund et al. (2021)’, second column should read: “Daily SMS-intervention over 5 consecutive competition days during the championship. The pilot phase was followed by three subsequent phases of development, which took place with 18 HPCs from athletics and figure skating. In each phase, HPCs completed a daily or weekly brief mindful self-reflection SMS-intervention for 8 weeks prior to taking part in a focus group interview and 6-month or 12-month follow-up.

Fidelity reported ranged from 92% - 100% in Phase 1, and 83% - 98% in phase 3.

Fidelity was reported to be 100% in Phase 2.”

instead of:

“Daily SMS-intervention over 5 consecutive competition days during the championship that yielded an 89% response rate, which provided a high enough fidelity and reach”. And the third column should read:

“Pilot: Daily SMS-intervention over 5 consecutive competition days during the championship that yielded an 89% response rate, which provided a high enough fidelity and reach.

Phase 1 - 3: SMS diary was trailed daily for 8 consecutive weeks. Reach reported ranged from 92% - 100% in Phase 1, and 83% - 98% in phase 3. Fidelity and reach were reported to be 100% in Phase 2.”

instead of:

“daily SMS-intervention over 5 consecutive competition days during the championship that yielded an 89% response rate, which provided a high enough fidelity and reach”.

The emendations have been made to the article.

