# Supplementary material for: Process evaluations of health-promotion interventions in sports settings: a systematic review
Source: Health Promot Int. 2023 Sep 18;38(5):daad114. doi: 10.1093/heapro/daad114 (PMC10506172; doi:10.1093/heapro/daad114)
Supplement: daad114_suppl_Supplementary_Material [file daad114_suppl_supplementary_material.pdf]

Electronic Supplementary File 1. PRISMA Checklist

PRISMA 2020 Checklist

Section and Topic	Item #	Checklist item	Location where item is reported
**TITLE**			
Title	1	Identify the report as a systematic review.	1
**ABSTRACT**			
Abstract	2	See the PRISMA 2020 for Abstracts checklist.	1
**INTRODUCTION**			
Rationale	3	Describe the rationale for the review in the context of existing knowledge.	2
Objectives	4	Provide an explicit statement of the objective(s) or question(s) the review addresses.	2
**METHODS**			
Eligibility criteria	5	Specify the inclusion and exclusion criteria for the review and how studies were grouped for the syntheses.	5
Information sources	6	Specify all databases, registers, websites, organisations, reference lists and other sources searched or consulted to identify studies. Specify the date when each source was last searched or consulted.	5-6
Search strategy	7	Present the full search strategies for all databases, registers and websites, including any filters and limits used.	5
Selection process	8	Specify the methods used to decide whether a study met the inclusion criteria of the review, including how many reviewers screened each record and each report retrieved, whether they worked independently, and if applicable, details of automation tools used in the process.	5-6
Data collection process	9	Specify the methods used to collect data from reports, including how many reviewers collected data from each report, whether they worked independently, any processes for obtaining or confirming data from study investigators, and if applicable, details of automation tools used in the process.	6-7
Data items	10a	List and define all outcomes for which data were sought. Specify whether all results that were compatible with each outcome domain in each study were sought (e.g. for all measures, time points, analyses), and if not, the methods used to decide which results to collect.	6-7
	10b	List and define all other variables for which data were sought (e.g. participant and intervention characteristics, funding sources). Describe any assumptions made about any missing or unclear information.	8
Study risk of bias assessment	11	Specify the methods used to assess risk of bias in the included studies, including details of the tool(s) used, how many reviewers assessed each study and whether they worked independently, and if applicable, details of automation tools used in the process.	7
Effect measures	12	Specify for each outcome the effect measure(s) (e.g. risk ratio, mean difference) used in the synthesis or presentation of results.	N/A
Synthesis methods	13a	Describe the processes used to decide which studies were eligible for each synthesis (e.g. tabulating the study intervention characteristics and comparing against the planned groups for each synthesis (item #5)).	6-7
	13b	Describe any methods required to prepare the data for presentation or synthesis, such as handling of missing summary statistics, or data conversions.	6-7
	13c	Describe any methods used to tabulate or visually display results of individual studies and syntheses.	6-7
	13d	Describe any methods used to synthesize results and provide a rationale for the choice(s). If meta-analysis was performed, describe the	6-7

Section and Topic	Item #	Checklist item	Location where item is reported
		model(s), method(s) to identify the presence and extent of statistical heterogeneity, and software package(s) used.	
	13e	Describe any methods used to explore possible causes of heterogeneity among study results (e.g. subgroup analysis, meta-regression).	6-7
	13f	Describe any sensitivity analyses conducted to assess robustness of the synthesized results.	6-7
Reporting bias assessment	14	Describe any methods used to assess risk of bias due to missing results in a synthesis (arising from reporting biases).	6-7
Certainty assessment	15	Describe any methods used to assess certainty (or confidence) in the body of evidence for an outcome.	6-7
**RESULTS**			
Study selection	16a	Describe the results of the search and selection process, from the number of records identified in the search to the number of studies included in the review, ideally using a flow diagram.	8
	16b	Cite studies that might appear to meet the inclusion criteria, but which were excluded, and explain why they were excluded.	S. file 8
Study characteristics	17	Cite each included study and present its characteristics.	S. file 5
Risk of bias in studies	18	Present assessments of risk of bias for each included study.	N/A
Results of individual studies	19	For all outcomes, present, for each study: (a) summary statistics for each group (where appropriate) and (b) an effect estimate and its precision (e.g. confidence/credible interval), ideally using structured tables or plots.	S. file 5
Results of syntheses	20a	For each synthesis, briefly summarise the characteristics and risk of bias among contributing studies.	9-13
	20b	Present results of all statistical syntheses conducted. If meta-analysis was done, present for each the summary estimate and its precision (e.g. confidence/credible interval) and measures of statistical heterogeneity. If comparing groups, describe the direction of the effect.	N/A
	20c	Present results of all investigations of possible causes of heterogeneity among study results.	9-13
	20d	Present results of all sensitivity analyses conducted to assess the robustness of the synthesized results.	N/A
Reporting biases	21	Present assessments of risk of bias due to missing results (arising from reporting biases) for each synthesis assessed.	N/A
Certainty of evidence	22	Present assessments of certainty (or confidence) in the body of evidence for each outcome assessed.	6-7
**DISCUSSION**			
Discussion	23a	Provide a general interpretation of the results in the context of other evidence.	13-17
	23b	Discuss any limitations of the evidence included in the review.	13-17
	23c	Discuss any limitations of the review processes used.	17-18
	23d	Discuss implications of the results for practice, policy, and future research.	13-17
**OTHER INFORMATION**			
Registration and protocol	24a	Provide registration information for the review, including register name and registration number, or state that the review was not registered.	5
	24b	Indicate where the review protocol can be accessed, or state that a protocol was not prepared.	5
	24c	Describe and explain any amendments to information provided at registration or in the protocol.	5
Support	25	Describe sources of financial or non-financial support for the review, and the role of the funders or sponsors in the review.	Declaration

Section and Topic	Item #	Checklist item	Location where item is reported
Competing interests	26	Declare any competing interests of review authors.	Declaration
Availability of data, code and other materials	27	Report which of the following are publicly available and where they can be found: template data collection forms; data extracted from included studies; data used for all analyses; analytic code; any other materials used in the review.	5 - 9

From: Page MJ, McKenzie JE, Bossuyt PM, Boutron I, Hoffmann TC, Mulrow CD, et al. The PRISMA 2020 statement: an updated guideline for reporting systematic reviews. BMJ 2021;372:n71. doi: 10.1136/bmj.n71

For more information, visit: http://www.prisma-statement.org/

Electronic Supplementary File 2. Search strategy for each electronic database

#	Academic Search Complete	PsycArticles	PsycInfo	SportDiscus	Medline	SCOPUS	Pub Med	ProQuest Central
2	TX (sport* OR athlet*	TX (sport* OR athlet*	TX (sport* OR athlet*	TX (sport* OR athlet*	TX (sport* OR athlet*	ALL (sport* OR athlet*)	ALL (sport* OR athlet*)	ft (sport* OR athlet*
	AND interven* OR workshop* OR program*	AND interven* OR workshop* OR program*	AND interven* OR workshop* OR program*	AND interven* OR workshop* OR program*	AND interven* OR workshop* OR program*	AND ALL (interven* OR workshop* OR program*)	AND (interven* OR workshop* OR program*)	AND ft (interven* OR workshop* OR program*)
	AND (“process evaluation”)	AND (“process evaluation”)	AND (“process evaluation”)	AND (“process evaluation”)	AND (“process evaluation”)	AND ALL (“process evaluation”)	AND (“process evaluation”)	AND ft((“process evaluation”))
3	S1 AND S2 AND S3	S1 AND S2 AND S3	S1 AND S2 AND S3	S1 AND S2 AND S3	S1 AND S2 AND S3	S1 AND S2	S1 AND S2 AND S3	S1 AND S2 AND S3
4	S1, S2, S3 Limit: Boolean/Phrase, Peer reviewed	S1, S2, S3 Limit: Boolean/Phrase, Peer reviewed	S1, S2, S3 Limit: Boolean/Phrase, Peer reviewed	S1, S2, S3 Limit: Boolean/Phrase, Peer reviewed	S1, S2, S3 Limit: Boolean/Phrase, Peer reviewed	S1, S2, S3 Limit: Document Type (Article, Article in Press)	S1, S2, S3 Article types: clinical study, clinical trial, journal article, observational study, randomized controlled trial, review, evaluation study	S1, S2, S3 Limit: Peer reviewed

Electronic Supplementary file 3. 14-items quality assessment Grant et al. (2013)

	**Checklist**	**Explanation/ Rationale**
1	Process evaluation was tailored to the trial. (1)	Process evaluations must have designed their investigation methods (i.e., research design, process evaluation framework, purpose of data collected), according to the specific research questions laid out within the intervention.
2	Process evaluation was clearly labelled. (1)	They have CLEARLY mentioned that they have conducted a process evaluation. This was one of our screening inclusion criteria.
3	Process evaluation clearly stated their purpose. (1)	Studies must:<br>1. State their original purpose and research questions<br>2. The processes being studied, and<br>3. An acknowledgement of what is not being evaluated.<br>Any changes to research questions during the study should be explicitly mentioned.
4	Process evaluation clearly reported if they were:<br>i.) pre-specified or post hoc (0.5)<br>ii.) why the selected timing was chosen (0.5)	i.) Did they intend to perform a process evaluation BEFORE the intervention, or did they only decide to do a process evaluation AFTER the intervention (e.g., Hunt et al 2020)? This needs to be made clear.<br>ii.) Did they state why they performed the process evaluation using the time period that they did? It could be as simple as, because this was during the research period, or because that is the best time to observe what the participants think of the program.<br>An example statement:<br>"Our sampling strategy ensured participants were able to reflect on the HOAM program when run completely, and as such, were viewed to be a rich source of data." - Wynters et al. (2021)
5	Process evaluations stated the choice of methods and justified them in terms of the stated aims of the evaluation. (1)	The rationale of the methods used. These include: study methods used (e.g., qual, quant, or mixed), or the philosophical positions used to guide the methods.<br><br>An example statement:<br>"This study adopted an interpretivist philosophy to understanding adolescents' knowledge, attitudes, and perceptions of HOAM (Guba & Lincoln, 1994). This approach is grounded in a relativist ontology..." - Wynters et al. (2021)
6	Process evaluations:<br><br>i.) summarised or referred to the main findings of the trial. (0.5)<br>ii.) trial and evaluation reports cross- referenced each other. (0.5)	i.) Some process evaluations are published alongside the outcome evaluation of the intervention (e.g., Santos et al., 2019; Panza et al., 2022). Often, however, process evaluations are reported independently (e.g. Wynters et al., 2021). In cases where process evaluations are reported on its own, there should be a brief explanation about what the intervention found. The quality of the explanation doesn't matter.<br>ii.) If the main findings were published elsewhere, this should be clearly mentioned.

7	The wider context in which the trial is being conducted was considered. (1)	Whether they have considered factors that can influence outcomes in sports settings (e.g., training time, the sport setting itself). You'd find that these studies are often explicit in the context they are evaluating (e.g., professional football clubs/ sports clubs), and consider unique factors that may impact on intervention delivery (e.g., training time).
8	Process evaluation was informed by an existing evaluation framework (e.g. RE-AIM, MRC, CPO). (1)	Did they use a recommendation/ guideline (e.g. Saunders et al. (2005), Grant et al. (2013) or a framework (e.g., MRC, RE-AIM)?<br><br>NOTE: Philosophical positions alone do not count. They are often guidance for the choice of methods used to conduct the evaluation, or perform the analysis.
9	Process evaluation considered whether the recruitment and reach of individuals is representative of the population. (1)	This considers how many clubs/participants they advertised to, and how many participants actually signed up for the program. Typically involves percentages, and you can find this in the demographic/ participant recruitment sections (if reported).<br><br>An example statement for considering reach:<br>"Among clubs contacted via e-mail or phone, 8% of clubs replied to recruitment with only 1% scheduling an intervention. In addition to tracking club-level adoption of the intervention approach, we also tracked involvement of coaches/teams within those club settings as well as athletes within teams. In total, seven clubs decided to adopt the intervention. Across these seven clubs, we had 73% adoption from specific teams..." - Panza et al. (2022)
10	Delivery of the intervention was considered in the process evaluation. (1)	Whether they have set out to observe the implementation of the intervention. Can include fidelity measures, observations, interviews, document logs, participant focus groups.
11	Process evaluation considered the response of individuals in the target population. (1)	They must have investigated the participants. Some studies only look at the delivery staff's perspective - in that case, this would be a 0, because that is not the target population.
12	Process evaluation considered:<br>i.) how the intervention processes was sustained over time (or not) (0.5)<br>ii.) why the intervention processes was sustained over time (or not) (0.5)	i.) Did they investigate the factors that led to the continued success of their intervention? This needs to be explicitly mentioned, and not implied. This can sometimes look like facilitators and barriers to intervention.<br>ii.) Did they investigate the factors that explained why the intervention was successful in the long term?
13	Process evaluation considered effects on:<br>i.) primary outcomes (0.5)<br>ii.) Secondary outcomes (0.5)	Did the process evaluation consider how the implementation process may have impacted on (or is associated with) the primary and secondary outcomes? This typically aims to help explain why an intervention did or did not work. Often, studies explore primary, but not secondary outcomes.
14	Process evaluation considered effects of unintended consequences. (1)	Did they set out to explore the unintended consequences/casual assumptions that emerge from the data? It can include clearly listing causal assumptions and testing for it. Often, this consideration also presents as things that both help AND hinder the

		implementation of an intervention. For example, Wynters et al (2021) found that whilst having a large group is good for the program, there needs to be more consideration for the group dynamics.

Electronic Supplementary File 4. Quality appraisal individual items scoring.

	**Items corresponding to quality appraisal checklist following Grant et al. (2013) – Supplementary file 8**																	
**Studies**	**1**	**2**	**3**	**4(i)**	**4(ii)**	**5**	**6(i)**	**6(ii)**	**7**	**8**	**9**	**10**	**11**	**12(i)**	**12(ii)**	**13(i)**	**13(ii)**	**14**	**Total**
**DeCelels et al. (2016)**	1	1	1	0	0.5	1	0.5	0.5	1	0	0	1	1	0.5	0	0.5	0	0	9.5
**Santos et al. (2019)**	1	1	1	0.5	0	1	0.5	0.5	1	0	0	1	1	0	0	0.5	0	0	9
**Maddison et al. (2019)**	1	1	1	0	0.5	1	0.5	0.5	1	0	1	1	1	0	0	0.5	0	0	10
**Parnell et al. (2015)**	1	1	1	0.5	0.5	1	0.5	0.5	1	1	1	1	1	0	0.5	0.5	0	0	12
**Newman et al. (2020)**	1	1	1	0	0	1	0.5	0.5	1	0	0	1	1	0.5	0	0.5	0	0	9
**Eather et al. (2021)**	1	1	1	0.5	0.5	1	0.5	0.5	1	0	0	1	1	0.5	0	0.5	0	0	10
**Hurley et al. (2018)**	1	1	1	0.5	0	1	0.5	0.5	1	0	0	1	1	0	0	0.5	0	0	9
**Wynters et al. (2021)**	1	1	1	0	1	1	0.5	0.5	1	1	0	1	1	0	0.5	0.5	0	1	12
**Fuller et al. (2015)**	1	1	1	0.5	0	1	0.5	0.5	1	0	0	1	1	0	0	0.5	0	1	10
**Roncarolo et al. (2015)**	1	1	1	0.5	0.5	1	0.5	0.5	1	1	1	1	1	0.5	0.5	0.5	0	0	12.5
**Mansfield et al. (2018)**	1	1	1	0.5	0	1	0.5	0.5	0	0	0	1	1	0.5	0	0.5	0	0	8.5
**Vella et al. (2019)**	1	1	1	0.5	0.5	1	0.5	0.5	1	1	1	1	1	0.5	0.5	0.5	0	0	12.5
**Hunt et al. (2014)**	1	1	1	0.5	0	1	0.5	0.5	1	0	0	1	1	0.5	0.5	0.5	0.5	1	11.5
**Rookhuijzen & DeVet (2021)**	1	1	1	0.5	0.5	1	0.5	0.5	1	0	1	1	1	0.5	0.5	0.5	0	0	11.5
**Naylor et al. (2015)**	1	1	1	0.5	0.5	1	0.5	0.5	1	0	0	1	1	0.5	0.5	0.5	0	0	10.5
**Gray et al. (2013)**	1	1	1	0.5	0.5	1	0.5	0.5	1	1	1	1	1	0.5	0.5	0.5	0.5	0	13
**Dunn (2004)**	1	1	1	0.5	0	1	0	0	1	0	0	1	1	0	0	0.5	0	1	9
**Robertson et al. (2013)**	1	1	1	0.5	0.5	1	0.5	0.5	1	0	1	1	1	0.5	0.5	0.5	0	1	12.5
**Lauwerier et al. (2020)**	1	1	1	0.5	0.5	1	0	0.5	1	1	0	1	1	0.5	0.5	0.5	0	0	11
**Hägglund et al. (2021)**	1	1	1	0.5	0.5	1	0.5	0.5	1	1	1	1	1	0.5	0	0.5	0	0	12

**Hunt et al. (2020)**	1	1	1	0.5	0.5	1	0.5	0.5	1	1	1	1	1	0.5	0.5	0.5	0	1	13.5
**Kwasnicka et al. (2021)**	1	1	1	0.5	0	1	0.5	0.5	1	1	1	1	1	0.5	0.5	0.5	0	1	13
**Eather et al. (2020)**	1	1	1	0	0	0	0.5	0.5	1	0	0	1	1	0	0	0.5	0	0	7.5
**Chen (2020)**	1	1	1	0.5	0.5	1	0.5	0.5	1	1	0	1	1	0	0	0.5	0.5	1	12
**McGregor (2019)**	1	1	1	0.5	0.5	1	0.5	0.5	1	1	0	1	1	0	0	0.5	0.5	1	12
	1	1	1	0.5	0.5	1	0	0	1	1	0	1	1	0	0	0.5	0.5	0	10
**Boelsen-Robinson et al. (2021)**	1	1	1	0.5	0.5	1	0.5	0.5	1	0	1	1	1	0	0	0.5	0	0	10.5
**Sandgren et al. (2021)**	1	1	1	0.5	0.5	1	0.5	0.5	1	1	1	1	1	0	0	0.5	0.5	0	12
**Petrella et al. (2022)**	1	1	1	0	0	1	0	0	1	1	0	1	1	0	0	0.5	0	0	8.5
**Waters et al. (2022)**	1	1	1	0.5	0	1	0	0.5	1	1	1	1	1	0	0	0.5	0	1	11.5
**Panza et al. (2022)**	1	1	1	0.5	0.5	1	0.5	0	1	1	1	1	1	0.5	0.5	0.5	0	0	12
**Brooke et al. (2022)**	1	1	1	0.5	0.5	1	0.5	0	1	1	0	1	1	0	0	0.5	0.5	1	11.5

Electronic Supplementary File 5. Characteristics of Process Evaluations, Including Main Intervention Description, Aims of the Process Evaluation, and Process Evaluation Measures of Included Studies.

**Study** *(Author, Year, Country)*	**Intervention Overview** *(Description, intervention duration, study design)*	**Process Evaluation study design** *(Design, measures, perspectives observed)*	**Process Evaluation participant characteristic** *(Age [mean and SD or range/median if available, N [if available], Gender [% male, if available], Sport [if available])*	**Process Evaluation Frameworks**	**Process Evaluation Outcomes tested**	**QA**
DeCelles et al. (2016)<br><br>Zimbabwe	Voluntary Male Medical Circumcision (VVMC) promotion intervention<br><br>x1 60-minute interactive, soccer-themed educational session with follow-up behavioral and logistical reinforcement<br><br>Cluster RCT	**Design:** Qualitative<br>**Measures:**<br>2 focus groups and in-depth interviews<br>**Perspectives observed:**<br>Participants (athletes)<br>Delivery staff (Coaches)	**Age:** Range 14-30<br>**N** = 46<br>**Gender:** Males (100%)<br>**Sport:** Soccer	N/A	Acceptability of intervention<br>Influential factors in deciding whether to VMMC (Adoption of intervention)<br>Fidelity<br>Effectiveness	9.5
Santos et al. (2019)<br><br>Portugal	Online PYD- focused coach education course	**Design:** Qualitative<br>**Measures:**<br>Reflective journal<br>Semi-structured interview<br>Field notes (observation) (N=2)<br>**Perspectives observed:**<br>Participants (Coaches)	**Age:** M age= 37 (SD = 12.8)<br>**N** = Not available for process evaluation<br>**Gender:** Males (71%)<br>**Sport:** Hockey, Equestrian	N/A	Fidelity<br>Effectiveness	9
Maddison et al. (2019)<br><br>New Zealand	RUFIT-NZ: gender sensitized weight loss program adapted from Football Fans in Training (FFIT) NZ delivered via professional rugby clubs<br><br>12 weeks, 2- arm RCT	**Design:** Quantitative<br>**Measures:**<br>Document logs<br>Participant feedback surveys<br>**Perspectives observed:**<br>Participants (Men)	**Age:** M age= 40.6 (SD=8.9)<br>**N** = 49<br>**Gender:** Males (100%)<br>**Sport:** Rugby	N/A	Reach<br>Effectivness/Acceptability (Survey, recruitment rates & retention)	10
Parnell et al. (2015)<br><br>UK	Extra Time (ET): national program of interventions delivered in/by Premier and Football League clubs for OA aged 55+ years<br><br>Duration:<br>2 years<br>Mixed methods	**Design:** Quantitative<br>**Measures:**<br>Self-report outcomes<br>Qualitative analysis (convenience sampling system)<br>**Perspectives observed:**<br>Participants (Older adults aged 55+)<br>Delivery staff (research team)	**Age:** Females (Median= 70–74 years); Males (Median= 65–69 years)<br>**N** = 486<br>**Gender**: Males (40.3%)<br>**Sport:** Mixed	RE-AIM	Reach<br>Adoption<br>Implementation<br>Fidelity<br>Effectiveness/Acceptability	12
Newman et al. (2020)	Sports-based PYD program designed for youth who are socially vulnerable.	**Design:**<br>Quantitative<br>**Measures:**	**Age:** Range (19- 27)<br>**N** = 26<br>**Gender:** Males (42.3%)	N/A	Adoption<br>Implementation<br>Fidelity	9

USA	19-day summer camp, organized into 15 days of curricula that built towards a 4-day culminating event. Each day of curricula involved four 60-minute sessions: three sport sessions designed to foster life-skill development by infusing life- and sport-skill instruction, and one classroom-based education session designed to support life- skill development through play-based activities.	Session logs<br>**Perspectives observed:**<br>Delivery staff (research team)	**Sport:** Basketball, dance, football, health and fitness, lacrosse, soccer, softball, swimming, and volleyball		Effectiveness/Acceptability	
Eather et al. (2021)<br><br>Australia	MASTER coaching program<br>8-week coach development intervention on game-based coaching practices of netball coaches<br>RCT	**Design:**<br>Quantitative<br>**Measures:**<br>Pre-post measures<br>Peer observation/open discussion<br>**Perspectives observed:**<br>Participants (coaches)	**Age:** M age = 45.0 (SD 8.30)<br>**N =** 16<br>**Gender:** Male (> 1%)<br>**Sport:** Netball	N/A	Fidelity<br>Feasibility<br>Implementation<br>Effectiveness<br>Recruitment<br>Retention<br>Adherence<br>satisfaction	10
Hurley et al. (2018)<br><br>Australia	No.1 Supporter, a parent mental health literacy program delivered in youth sport clubs designed to raise awareness of parents' role in promoting and supporting positive adolescent mental health and increase parental mental health literacy.<br>x1 hour workshop, matched control design	**Design:**<br>Mixed methods<br>**Measures:**<br>Open ended feedback<br>Follow up semi-structured interviews & focus groups<br>**Perspectives observed:**<br>Participants (Parents)	**Age:** M age = 44.86 ± 5.2 years<br>**N =** Quantitative (*n*=44); Qualitative (*n*=4)<br>**Gender:** Males (33%)<br>**Sport:** Not available	N/A	Acceptability<br>Feasibility<br>Effectiveness	9
Wynters et al. (2021)<br><br>Australia	Help Out a Mate: Sports-based mental health literacy intervention aimed to raise mental health literacy among male adolescents in community sports clubs.	**Design:**<br>Qualitative<br>**Measures:**<br>Focus groups (6 groups)<br>**Perspectives observed:**<br>Participants (athletes)	**Age:** Range (12-15) (M = 13.79, SD = 0.91)<br>**N =** 33<br>**Gender:** Males (100%)<br>**Sport:** Soccer	MRC	Implementation<br>Fidelity<br>Effectiveness<br>Acceptability	12
Fuller et al. (2014)<br><br>UK	SPORTSMART, intervention aimed at improving STI screening.<br><br>Pilot cluster-RCT	**Design:**<br>Mixed methods<br>**Measures:**<br>Field notes<br>Measures (self-report)<br>Semi-structured interviews<br>**Perspectives observed:**<br>Delivery staff (captains and sexual health advisers)<br>Participants (semi-structured IV)	**Age:** Unclear<br>**N=** 183<br>**Gender:** Males (100%)<br>**Sport:** Football	N/A	Feasibility<br>Fidelity<br>Effectiveness<br>Acceptability	10
Roncarolo et al. (2015)<br><br>UK, Finland, Greece, Ireland,	Healthy sports stadia program, aimed at supporting health improvement and reduce health inequalities through interaction with local communities and with the people who use the stadia as a place to practice sport, for entertainment or work.	**Design:**<br>Quantitative<br>**Measures:**<br>Questionnaires<br>**Perspectives observed:**	**Age:** Not available for process evaluation<br>**N =** 9<br>**Gender:** Males	Issel (2004)	Feasibility<br>Fidelity<br>Reach<br>Implementation<br>Effectiveness	12.5

Italy, Latvia, Poland, and Spain.		Participants (club stakeholders)	**Sport:** Football, but basketball, rugby, volleyball, cricket and multi-sport stadia, along with minor sport stadia such as greyhound racing, also participated in the program.			
Mansfield et al. (2018)<br><br>UK	Engage previously inactive people in sustained sporting activity for 1×30 min a week, examine the associated health and wellbeing outcomes of doing so, and produce information of value to those commissioning public health programmes that could potentially include sport.<br>Mixed methods study	**Design:**<br>Qualitative<br>**Measures:**<br>Semi-structured interview<br>**Perspectives observed:**<br>Participants (Coaches)	**Age:** Not available for process evaluation<br>**N** = 32<br>**Gender:** Males<br>**Sport:** Not available	N/A	Implementation<br>Participant experiences	8.5
Vella et al. (2019)<br><br>Australia	Ahead of the Game, multi-component program targeting distinct levels of influence regarding adolescent mental health through sport.<br><br>RCT	**Design:**<br>Mixed<br>**Measures:**<br>Extended consultation w stakeholders<br>**Perspectives observed:**<br>Participants (Parents, coaches, adolescents)<br>Delivery staff (recruitment logs)	**Age:** Not available for process evaluation<br>**N** = Phase 1 not available for process evaluation; Phase 2: Adolescents (n=167); Parents (n=64); Coaches (n=1)<br>**Gender:** Males (100%)<br>**Sport:** Mixed	CFIR<br>NIRN<br>Saunders et al. (2005)	Feasibility of intervention<br>Implementation<br>Barriers to implementation<br>Reach<br>Fidelity<br>Effectiveness/ Acceptability<br>Maintenance	12.5
Hunt et al. (2014)<br><br>UK	Football Fans in Training (FFIT)<br>12-session, weight management and physical activity group programme with subsequent minimal-contact weight loss maintenance support delivered free of charge	**Design:**<br>Mixed Methods<br>**Measures:**<br>Focus groups<br>Baseline measures<br>**Perspectives observed:**<br>Participants (men)	**Age:** Range (35-65)<br>**N** = 63<br>**Gender:** Males (100%)<br>**Sport:** Soccer	N/A	Participant characteristics<br>Participant motivation for engagement	11.5
Rookhuijzen & DeVet (2021)<br><br>Netherlands	Nudge intervention implemented to promote healthy eating in two football canteens.<br><br>Within sample, mixed method study	**Design:**<br>Mixed Methods<br>**Measures:**<br>Questionnaires<br>Semi-structured interviews<br>Observations<br>**Perspectives observed:**<br>Board members<br>Delivery staff (canteen staff)	**Age:** Not available for process evaluation<br>**N** = 4<br>**Gender:** Males<br>**Sport:** Football	N/A	Reach<br>Acceptability<br>Adherence (via random visit observations)<br>Applicability	11.5
Naylor et al. (2015)<br><br>Canada	Capacity-building intervention (Healthy Food and Beverage Sales; HFBS) on organizational capacity for providing healthy food environments, health of vending machine products, and food policy development in recreation and sport facilities	**Design:**<br>Mixed Methods<br>**Measures:**<br>In-depth examination of Project proposals	**Age:** Not available for process evaluation<br>**N** = Not available for process evaluation<br>**Gender:** Males	N/A	Implementation<br>Facilitators and barriers<br>Utilisation of skills<br>Effectiveness<br>Intervention impact	10.5

	Quasi-experimental, controlled,<br>pre-post comparison design natural experiment large, controlled study	Final reports<br>Semi structured telephone Interviews conducted with key recreation staff at follow-up<br>**Perspectives observed:**<br>Delivery staff (research team)	**Sport:** Ice arenas, curling rinks, pools, fitness gyms, outdoor sporting facilities, and multiplexes that housed several of these within a single facility			
Gray et al. (2013)<br><br>UK	Football Fans in Training (FFIT)<br>12-session, weight management and physical activity group programme with subsequent minimal-contact weight loss maintenance support delivered free of charge<br>RCT	**Design:**<br>Mixed Methods<br>**Measures:**<br>Qualitative - Focus groups<br>Quantitative - Post program questionnaires<br>**Perspectives observed:**<br>Participants<br>Coaches<br>Participants exit reasons!	**Age:** 34+<br>**N =** Quantitative (*n*=155); Qualitative (*n*=39)<br>**Gender:** Males (100%)<br>**Sport:** Football	MRC	Reach<br>Implementation<br>Fidelity<br>Effectiveness<br>Acceptability<br>Program components<br>Exit reasons	13
Dunn et al. (2004)<br><br>Canada	Personal-Disclosure Mutual-Sharing (PDMS) team building activity delivered at a national championship tournament where the team was required to play three games in a four-day period.	**Design:**<br>Qualitative<br>**Measures:**<br>Semi structured interviews<br>**Perspectives observed:**<br>Participants	**Age:** M age = 22.4 years (SD = 1.4)<br>**N=** 27<br>**Gender:** Males (100%)<br>**Sport:** Ice hockey	N/A	Participants' subjective perceptions<br>Program component	9
Robertson et al. (2013)<br><br>UK	Premier League Health (PLH), a three-year programme of men's health promotion located in 16 top-flight English football clubs targeted men aged between 18 and 35years from socio-economically deprived communities.	**Design:**<br>Qualitative<br>**Measures:**<br>Semi structured interviews<br>**Perspectives observed:**<br>Delivery staff (research team) n=16<br>Participants (men) n= 58	**Age:** Range (18 - 35 years)<br>**N =** Not available for process evaluation<br>**Gender:** Males (100%)<br>**Sport:** Football	N/A	Feasibility<br>Effectiveness<br>Reasons for engagement with the initiative<br>Reasons for staying involved<br>Reach	12.5
Lauwerier et al. (2020)<br><br>Belgium	Sport for development (SFD) training that targeted coaches to improve their knowledge and skills for the transfer of health promotion messages to at-risk youth.	**Design:**<br>Mixed Methods<br>**Measures:**<br>Document logs<br>Direct observations of intervention delivery n=10<br>Session evaluation questionnaires (for participants and staff)<br>Semi-structured interviews (with participants and staff) n=8<br>Focus groups (staff and key stakeholders) n=8<br>**Perspectives observed:**<br>Participants (SFD coaches)	**Age:** M age= 30.4<br>**N =** 8<br>**Gender:** Males (100%)<br>**Sport:** Not available	Medical Research Council	Feasibility<br>Theory of change<br>Central mechanisms of impact<br>Reach<br>Adoption<br>Implementation<br>Effectiveness/Acceptability<br>Maintenance	11

		Delivery Staff<br>Stakeholders (local policy makers)				
Hägglund et al. (2021)<br><br>Sweden	Mindfulness self-reflection intervention (delivered via SMS) to support sustainable high-performance coaching.	**Design:**<br>Qualitative<br>**Measures:**<br>SMS- diaries, focus group interviews and written follow-ups<br>**Perspectives observed:**<br>Participants (High performance coaches)	**Age:** Range (31-62)<br>**N** = 17<br>**Gender:** Males (38.8%)<br>**Sport:** Phase 1 & 2 not available; Phase 3: aesthetic sport, figure skating	Saunders et al. (2005)	Fidelity<br>Reach<br>Perceived value of intervention<br>Key mechanisms driving intervention<br>Behavioral changes	12
Hunt et al. (2020)<br><br>Australia, Canada, New Zealand, England and other European countries	Football Fans in Training (FFIT)<br><br>12 weekly group-based sessions (90-min) delivered free of charge to participants by community coaches within professional football clubs.<br>RCT	**Design:**<br>Mixed<br>**Measures:**<br>PRACTIS guide<br>**Perspectives observed:**<br>**Unclear**	**Age:** M age= 47.6 years (SD 9.5)<br>**N** = Not available for process evaluation<br>**Gender:** Males (100%)<br>**Sport:** Football	PRACTIS Guide<br>Koorts et al.'s (2019)<br>Aarons et al.'s distinction between 'scaling up' and 'scaling out'<br>RE-AIM (previous)	Implementation<br>Identification and engagement with key stakeholders<br>Contextual facilitators and barriers<br>Reach<br>Adoption<br>Implementation<br>Fidelity<br>Effectiveness/Acceptability<br>Maintenance	13.5
Kwasnicka et al. (2022)<br><br>Australia	Football Fans in Training (FFIT)<br><br>12 weekly group-based 90-min sessions, delivered free of charge to participants by community coaches within professional football clubs<br><br>RCT	**Design:**<br>Mixed Methods<br>**Measures:**<br>Participants focus groups (6 groups, n= 24): for both IV and C<br>Coach interviews (n=4) (coach delivered)<br>Audio recordings of Aussie-FIT sessions<br>Post-program participant surveys (n= 93)<br>**Perspectives observed:**<br>Participant<br>Delivery staff (Coaches)	**Age:** M age= 51.05 (SD = 10.09)<br>**N**= 24<br>**Gender:** Males (100%)<br>**Sport:** Football	Medical Research Council	feasibility and accept- ability of program setting and context, recruitment strategies, factors impacting implementation and mechanisms of impact.	13
Eather et al. (2020)<br><br>Australia	MASTER coaching program<br><br>8-week coach development intervention on game-based coaching practices of netball coaches<br><br>Quantitative	**Design:**<br>Quantitative<br>**Measures:**<br>Satisfaction data (short evaluation questionnaire)<br>**Perspectives observed:**<br>Participants (Coaches delivering MASTER)	**Age:** Not available for process evaluation<br>**N** = 6<br>**Gender:** Males (80%)<br>**Sport:** Football	N/A	Recruitment<br>Retention<br>Adherence<br>Satisfaction	7.5

Chen (2020)<br><br>Canada	Transformational coaching workshop to improve coaching behaviors and facilitate athletes' quality experiences in para-sport<br><br>Mixed methods, one arm	**Design:**<br>Mixed Methods<br>**Measures:**<br>Questionnaire<br>Semi-structured interview<br>Fidelity checklist<br>**Perspectives observed:**<br>Participants (coaches)	**Age:** M age = 41.63 (SD = 17.24)<br>**N =** 17<br>**Gender:** Males (41%)<br>**Sport:** Para-sport	Rogers' diffusion of innovation theory (DOI)	Experiences and development as a coach<br>Questions related to DOI theory and other factors that describe the workshop delivery + coaches' thoughts on principles of transformational coaching<br>Program perception<br>Fidelity (fidelity checklist)	12
McGregor (2019)<br><br>UK	Applied-practice intervention that focused on adolescent emotional development and emotion regulation.<br><br>Mixed methods, Action research approach	**Design:**<br>Mixed methods<br>**Measures:**<br>Surveys<br>Observations<br>Interviews<br>**Perspectives observed:**<br>Participants<br>(Coaches, *n*=11; Players, *n*= 13)	**Age:** Not available for process evaluation<br>**N** = 24<br>**Gender:** Males (100%)<br>**Sport:** Football	Nielsen et al. (2010)	Implementation<br>Fidelity<br>Effectiveness<br>Acceptability<br>Utilisation of skills	12
O'Brien et al. (2021)<br><br>Canada	Develop and implement IPEP, a tailored injury prevention program in soccer teams	**Design:**<br>**Unclear**<br>**Measures:**<br>semi structured interviews (staff members)<br>Focus groups (layers)<br>**Perspectives observed:**<br>Participants (38 players and staff members)	**Age:** Not available in text<br>**N** = 22<br>**Gender:** Males (100%)<br>**Sport:** Soccer	Consolidated Framework for Implementation Research (CFIR)<br>Medical Research Council	Implementation<br>Barriers and Facilitators<br>Performance outcomes<br>Reach<br>Dose	10
Boelsen-Robinson et al. (2021)<br><br>Australia	Sugar-sweetened beverage (SSB) nudge intervention in community sport clubs can help promote healthy eating behaviors.	**Design:**<br>Observational<br>**Measures:**<br>Semi structured interviews (staff members)<br>Focus groups (Participants)<br>**Perspectives observed:**<br>Customer (Participants)	**Age:** Not available for process evaluation<br>**N =** 806<br>**Gender:** Not available for process evaluation<br>**Sport:** Aquatic and recreation center	N/A	Fidelity<br>Implementation<br>Customer acceptability<br>Adoption	10.5
Sandgren et al. (2022)<br><br>UK	10-week novel Motivational and Psycho-Educational Self-Help Programme for Athletes with Mild Eating Disorder Symptoms (MOPED-A)<br><br>Intervention-arm only, Mixed methods	**Design:**<br>Mixed Methods<br>**Measures:**<br>Self-report outcomes by athletes<br>Interviews (n=15)<br>**Perspectives observed:**<br>Participants (athletes)	**Age:** M age = 27.1 years (SD = 8.2)<br>**N** = 15<br>**Gender:** Males (23.5%)<br>**Sport:** Any sport, at any level	Bowen et al. (2009)	Recruitment<br>Retention<br>Acceptability<br>Satisfaction<br>Program component<br>Feasibility	12
Petrella et al. (2022)	Supportive care service designed to engage testicular cancer survivors in supportive care by leveraging a community-based sport and exercise model aimed at supporting long-term survivorship	**Design:**<br>Mixed Methods<br>**Measures:**	**Age:**<br>Mage = 32.7 (SD = 6.41)<br>**N** = 10<br>**Gender:** Males (100%)	N/A	Satisfaction (acceptability)	6.5

Canada	outcomes in men living with and beyond testicular cancer.<br><br>Weekly (2-h session), lasting 5-weeks	Open ended feedback surveys (participants)<br>Researcher field notes<br>Focus groups with intervention participants<br>**Perspectives observed:**<br>Participants (Men)<br>Delivery staff (Research team)	**Sport:** Basketball			
Waters et al. (2022)<br><br>Australia	'Life-Fit-Learning system' designed to assess the mental health and wellbeing of youth (Assess step), provide feedback on assessment results (Reflect step), and connect them to resources and services proportionate to their needs (Connect step).	**Design:**<br>Mixed Methods<br>**Measures:**<br>Feasibility rating framework by research team (reports of obstacles)<br>Acceptability (youth satisfaction ratings)<br>**Perspectives observed:**<br>Participants (Athletes)<br>Delivery staff (Research team)	**Age:** Not available for process evaluation<br>**N =** 173<br>**Gender:** Males (100%)<br>**Sport:** Rugby	Proctor et al.'s framework of implementation outcomes	Feasibility (ease of implementation)<br>Penetration (degree of integration within each setting)<br>Acceptability (stake- holder and consumer satisfaction)	11
Panza et al. (2022)<br><br>USA	Team Talk intervention–a brief workshop-based intervention to increase adolescent athletes' mental health literacy while linking such messages to group norms and identities.	**Design:**<br>Mixed Methods<br>**Measures:**<br>Recruitment and implementation logs<br>Surveys<br>Interviews<br>**Perspectives observed:**<br>Participants (Athletes, *n*=5)<br>Stakeholders (Parents, *n*=9; Coaches *n*=2)<br>Delivery staff (logbook)	**Age:** M age= 14.89 (SD = 1.36)<br>**N =** 16<br>**Gender:** Males (16%)<br>**Sport:** Lacrosse, hockey, wrestling	RE-AIM Framework	Indices that relate to feasibility:<br>Reach<br>Implementation<br>Adoption<br>Maintenance<br>Effectiveness (proximal mechanisms)	14
Brooke et al. (2022)<br><br>Australia	First Episode Psychosis (FEP) recovery intervention<br><br>6-weeks sport programme alongside participant's support workers (i.e., community and peer workers) from the service, including peer workers with a lived experience of psychosis.	**Design:**<br>Mixed Methods<br>**Measures:**<br>qualitative data and compared this information with other data collected (e.g., attendance, feedback, quantitative measurements)<br>Semi structured interview<br>**Perspectives observed:**<br>Participants (*n*=3)<br>Stakeholders (Staff accompanying participants, *n*=5)	**Age:** Range (15–25 years)<br>**N =** 7<br>**Gender:** Males (100%)<br>**Sport:** Mixed	Medical Research Council<br>Bowen et al. (2009) framework for feasibility studies	Feasibility<br>Intervention components<br>Secondary outcomes<br>Facilitator and barriers	12

Electronic Supplementary file 6. Description of Intervention Delivery, Adjustments to Intevention Delivery Reported, and Expected Mechanisms of Impact of Included Studies.

**Study**	**Intended delivery of main intervention** *(Main intervention aim, detailed main intervention description, detailed main intervention procedure)*	**Actual delivery** *(Reported difference from intended delivery)*	**Expected mechanism of impact** *(Theories underpinning intervention, rationale behind how main intervention is theorised to work)*
DeCelles et al. (2016)	"Make the Cut" (MTC) and "Make the Cut+" (MTC+) - 2 brief and scalable voluntary medical male circumcision (VMMC) promotion interventions for males in Bulawayo, Zimbabwe.<br><br>60-minute educational sessions comprised 3 activities: "Cut and Cover," a soccer-based activity with known effectiveness in increasing knowledge of the health benefits of VMMC12; "Coach's Story," a motivating personal story from the facilitator about his experience undergoing and recovering from VMMC; and a question-and-answer period.	(Adjustments)<br>Two cluster RCTs were conducted to evaluate whether the interventions increased VMMC uptake among intervention participants.<br><br>Revised version of the intervention developed in 2013, MTC+, consisted of the same 60-minute educational session but incorporated different forms of behavioural and logistical reinforcement: (1) phone-based follow-up by coaches to arrange transport to the VMMC clinic; (2) coach accompaniment to the VMMC clinic; and (3) soccer-based incentives valued at USD 5, which were offered to participants on VMMC completion. VMMC completion was determined by participants showing GRS coaches a stamped GRS referral card. VMMC clinic staff stamped the card if the participant completed the VMMC procedure at the clinic. 13 MTC+ was delivered in Bulawayo secondary schools to boys ages 14–19 by circumcised GRS coaches. A similar TOC was conducted, with an additional day for a storytelling workshop.<br><br>Intervention was modified in April 2014 as PSI requested that all incentives be removed from the intervention for concerns that the incentives were unsustainable. Incentives were only offered to participants in 5 of the 13 intervention schools.<br><br>Observation implementation showed that coaches sometimes skipped Cut and Cover with older participants (ages 30 and older), as the older participants did not want to participate in the activity, while MTC+ coaches consistently followed the steps in the curriculum.	Social learning theory: 60-minute session was delivered by circumcised GRS "coaches," ages 18–30; use of role models has been identified as a key facilitator of VMMC uptake.<br><br>Ideal platform and setting of intervention.
Santos et al. (2019)	A positive youth development-focused online coach education course. Program aimed to expose youth sport coaches to PYD principles and reflect it in their coaching.	No adjustments reported.	Positive youth development (PYD) framework<br><br>Social learning theory: Delivered in real-time by trained facilitators in ways that allow coaches to

	Delivered online in Portuguese from February 17th, 2016, to March 16th, 2016 (no session on March 2nd as coaches were not available). The course was synchronous and divided into eight three-hour sessions delivered twice a week on Mondays and Wednesdays (total of 24 hours of course time).<br><br>Eight sessions were deliberately structured to cover five main areas: (a) defining PYD and life skills, (b) designing sport programs conducive to PYD, (c) integrating PYD-based strategies, (d) assessing PYD- based interventions, (e) final considerations on the value of the course and lessons learned. Guest lecture in English for two hours (sessions one and seven respectively).		interact and exchange with one another (Newman et al., 2016).
Maddison et al. (2019)	RUFIT-NZ, an adaptation of the Football Fans in Training program delivered in Premier-league sports clubs in the UK. Two-arm parallel design pilot RCT<br><br>RUFIT-NZ was delivered via professional rugby clubs can help overweight or obese men make healthier lifestyle changes.<br><br>A total of 96 participants (n=49 intervention; n=47 controls) were recruited (n = 46 Auckland; n = 50 Dunedin). The intervention was delivered through professional rugby clubs and involved physical activity training and classroom sessions on healthy lifestyle behaviours.<br><br>12-week, twice-weekly 90-min sessions in Auckland (one during the weekend and one during the working week) and a once-weekly 120–150 min session in Dunedin (held early evening during the working week), which were run at the respective rugby clubs by a RUFIT-NZ coach. In Auckland, the weekend session consisted of a 30-min classroom session followed by a 60-min physical activity session, while the session during the working week consisted of a 90-min physical activity session only. In Dunedin, the session comprised 60min of physical activity and 60–90 min for the classroom session. The number of classroom sessions delivered was the same for the two clubs. The content of the classroom sessions was standardized, so that the men participating at both clubs received the same educational material.	(Adjustments)<br>Following on findings from the focus groups, the program aimed to focus less on weight, but have a more holistic approach targeting a range of health-related behaviours (nutrition, physical activity, sleep, and sedentary behaviour), thus classroom sessions covered information on SMART goal setting and other behaviour change strategies.	Gender sensitised weight loss approach: men were attracted to a program that was tailored for them and did not require extensive time commitments. They also valued education about energy balance and the use of humour to deliver simple messages.<br><br>Program inspired by FFIT, but conducted through professional rugby clubs, could be effective and feasible for targeting overweight and obese males in NZ. The program was named Rugby Fans in Training NZ (RUFIT-NZ). Rugby (Union and League) is an integral part of NZ culture, the most popular spectator team sport, with high participation rates, particularly among Māori and Pacific peoples. A gender-sensitive life- style intervention program that harnesses the popularity of rugby and the culture of 'male masculinity' surrounding it, may therefore help target this underserved group by addressing perceived barriers to participation (e.g., pre- occupations with weight loss and dieting being women's issues). Further, this approach capitalizes on the traditional male sporting environment, the powerful social and psychological connection to the sports team (e.g., loyalty, identity) that being a fan creates, and the opportunity for men-only support.

Parnell et al. (2015)	Extra Time (ET): national program of interventions delivered in/by Premier and Football League clubs for OA aged 55+ years. Centred on physical activity based social inclusion/ health-improvement activities and were delivered free of charge over two-years.<br><br>2 hour-long weekly classes and groups involving physical and social activities delivered free to participants. A broad menu of PA opportunities were offered, including exercise to music, indoor bowls, cricket, new age curling, walking football, alongside traditional board games, bingo, table tennis, Zumba and skittles, as seen elsewhere.<br>Endorsing the social component of PA, classes provided participants with an opportunity to socialise before, during and after sessions. Light refreshments were often provided after activities, while more formal, organised social events including day trips were also provided. Activities were commonly delivered within the stadia or club facilities. While outreach work has been recommended, the football club and stadia - which are trusted, iconic and community embedded facilities - were most commonly used.	No adjustments reported.	Since professional football clearly connects with groups unreached by conventional health approaches, it may create an important pathway for health improvement among these groups.
Newman et al. (2020)	Sports-based PYD program designed for youth who are socially vulnerable. 19-day summer camp, the program was organized into 15 days of curricula that built towards a 4-day culminating event. Each day of curricula involved four 60- minute sessions: three sport sessions designed to foster life-skill development by infusing life- and sport-skill instruction, and one classroom-based education session designed to support life- skill development through play-based activities.<br><br>Operated at a large Midwestern university in the United States. All procedures were approved by the Institutional Review Board at the university. The community sport-based PYD summer camp, LiFEsports Summer Camp, integrated key PYD program design features with sport-based activities as a way to promote life-skill development of youth participants. The program was created for youth in the local community, many of whom were youth of colour from economically disadvantaged communities.<br>Adult program staff were responsible for fostering a caring climate, using curricula to teach sport sessions, and providing structured instruction and facilitation. Each of these programmatic design features was created to	Quantitative data reported.<br><br>Program staff practices:<br>Perceived implementation scores were highest in program climate (M = 90.15, SD = 8.64), followed by program instruction (M = 84.91, SD = 11.88), and curriculum (M = 83.73, SD = 13.91). Among the individual items, those with higher perceived implementation included “Materials and supplies ready for when youth arrive” (M = 3.77, SD = 0.08), “Youth greeted as they arrive” (M = 3.76, SD = 0.08), and “Encouragement for participation by all” (M = 3.71, SD = 0.11), all reflecting the program climate. Items with lower levels of implementation included: “Preview of tomorrow’s life skill; sport skill” (M = 2.62, SD = 0.43; M = 2.92, SD = 0.53 respectively), and “Review of previous day sport skill” (M = 3.01, SD = 0.91), which all reflect program instruction.<br><br>Program climate:<br>high self-reported implementation and the program curriculum items had moderate implementation, a	Social vulnerability theory, youth who are socially vulnerable are recognized as being confronted with and experiencing a culmination of negative risk factors that impede their overall healthy development, such as systemic racism, poverty, and neighbourhood disorder (Vettenburg, 1998).<br><br>Using sport as a context for learning.

	intentionally promote the development and transfer of key life skills, including self-control, effort, teamwork, and social responsibility.	pattern emerged among the program instruction items. That is, implementation of the items reflecting instruction of the present day's sport and life skill were relatively high (mean scores ranging 3.49 to 3.63), whereas items reflecting review of past or preview of future sport/life skills had low to moderate implementation (mean scores ranging from 2.62 to 3.01).<br><br>Type of sport:<br>no significant differences in staff's reported implementation of the program climate (U = 84.00, z = 1.70, p = .096, r = 0.33), program curriculum (U = 67.00, z = 0.57, p = .601 r = 0.11), and program structure (U = 60.50, z = 0.13, p = .896, r = 0.01) across contact and non-contact sports (Table 1). Although not statistically significant, higher perceptions of program implementation were consistently reported for non-contact sports compared to contact sports. For both contact and non-contact sport activities, program climate had the greatest degree of perceived implementation, whereas program curriculum the lowest degree of perceived implementation.<br><br>Setting of sport:<br>significant difference in staff's reported implementation of the program climate (U = 12.00, z = -3.45, p < .001, r = 0.68) and program curriculum (U = 32.00, z = -2.29, p = .022, r = 0.46) across indoor and outdoor facilities, with program implementation significantly higher when sessions were implemented indoors (Table 1). However, there was no statistically significant difference in staff's self-reported implementation of the program instruction (U = 38.50, z = -1.91, p = .06, r = 0.37) across indoor and outdoor facilities. Program climate had the greatest degree of perceived implementation for indoor activities, whereas program instruction had the greatest degree of perceived implementation for outdoor activities; program curriculum had the lowest degree of perceived implementation for both settings.	
Eather et al. (2021)	MASTER coaching program, randomized controlled trial	No adjustments reported.	The core pillar of MASTER is 'positive coaching,' delivered through games-based coaching practices

	8-week coach development intervention on game-based coaching practices of netball coaches (n= 16; 8 intervention, 8 active-control; NSW Australia).<br><br>Program targets six core elements of sports coaching (i.e., the six letters of MASTER) (detailed in Table 1) Following baseline assessments, MASTER was implemented using a coach learning process involving three phases (commencing April 2018): (i) The MASTER Coach Development Workshop, (ii) Mentoring, and (iii) Coach Assessment and Reflection.<br><br>Phase 1 (MASTER Coach Development Workshop): Coaches participated in a 6-hour theory and practical face-to-face coach education workshop held at the University of Newcastle conducted by a member of the research team (NE). A combination of lecture, discussion and group work activities provided coaches with the theoretical underpinnings and practical applications of the MASTER elements and provided coaches with opportunities to plan and assess sessions based on the MASTER framework and MASTER evaluation tool. Coaches were provided with a PowerPoint presentation, printed course booklet, MASTER checklist, printed (and explained) examples of netball training activities and planning tasks.<br><br>Phase 2 (On-line Mentoring/Discussion):<br>Immediately following Phase 1, this 8-week phase involved coaches implementing MASTER elements in their normal 1hr weekly training session and the research team providing on-going support, facilitating discussion and providing training activity ideas via an on-line Facebook group page.<br><br>Phase 3 (Assessment and Reflection Sessions): At week four of the intervention (alongside Phase 2), the mentor (NE) prepared and implemented a mid- intervention one-hour netball training session for the coaches designed to reinforce all aspects of the MASTER framework. Coaches were involved in evaluating the session using the MASTER observation checklist (see supplemental file: Figure 1), and a group discussion was facilitated at the end of the session (approximately 45min). coaches continued implementing MASTER strategies in their normal training sessions and		taking a positive pedagogical approach and through game based practice, mistakes provide opportunities for players to learn (rather than being used as controlling devices).20,21 Using a positive view of mistakes in the learning process the coach facilitates both short and long-term player development, positive affective responses to participation, and the desire to remain in the sport.53,54 Creating supportive sporting environments that promotes social support for player learning and development also encourages all players (in an inclusive environment) to try hard in a climate of mutual respect.<br><br>Games based approach: games-based training sessions are more enjoyable - which is directly linked to player motivation, engagement and retention in sport.<br><br>Through player-centred, inquiry-based approaches to coaching (i.e., game-based coaching), and a deep understanding of the game, coaches can develop game-play skills and abilities, increase player engagement and motivation, develop positive relationships between coach and players, and among players, and provide positive affective experiences of learning through sport.48 In this approach, coaches should include dialogue/discussion, reflection and purposeful game play, and set high and explicit expectations to promote deep understanding of the game and facilitate active learning.

	were required to undertake two peer observations of a colleague (using the MASTER observation checklist).		
Hurley et al. (2018)	No.1 Supporter, a parent mental health literacy program delivered in youth sport clubs designed to raise awareness of parents' role in promoting and supporting positive adolescent mental health and increase parental mental health literacy.<br><br>x1 hour workshop<br><br>Matched control design	No adjustments reported.	Family and parent-focused mental health intervention programs are not widely available and have traditionally suffered from low participation rates.<br><br>The potential benefits of youth sport participation for mental health are well documented (Eime et al., 2013) but few sport organizations engage in mental health initiatives (Liddle et al. 2016). However, a previous study has shown that the mental health literacy of adult leaders in youth sport, such as coaches and parents, can be improved (Bapat et al., 2009). By aligning mental health promotion with physical health promotion, through the medium of a com- munity sport club, it might become easier to facilitate conversation around mental health, reduce stigma, and positively influence mental health knowledge and attitudes (Anwar-McHenry et al., 2012).
Wynters et al. (2021)	Help Out a Mate: Sports-based mental health literacy intervention aimed to raise mental health literacy among male adolescents in community sports clubs<br><br>Cluster-RCT<br><br>45-min mental health literacy workshop for adolescent males aged 12–18, designed to increase young males' knowledge about depression and anxiety, confidence and intentions to help someone else who may be experiencing a mental health problem, and decrease stigmatising attitudes towards mental health. HOAM utilises sport specific examples and themes throughout the workshop, including elite athlete examples, how mental health may present within sport, and help-seeking options within sport; as well as role-plays to practice the skills learned. HOAM is designed to be delivered to younger ages than existing youth mental health literacy programs, an important difference given that half of all mental health problems have their onset before the age of 14 (Kessler et al., 2005).<br><br>Delivered face-to-face in the clubhouse or changing rooms of all participating teams. The program was delivered by	No adjustments reported.	Sport has been identified as a vehicle for mental health promotion and early intervention (Liddle et al., 2017). Seventy-four percent of young people engaged in organised sport nationwide in 2015 (Australian Bureau of Statistics, 2015b). The organised sport environment facilitates mental health promoting behaviours and can provide support networks and resources (Vella et al., 2017).

	members of the research team with training in MHFA and experience delivering sport-based mental health programming, and in some cases, alongside a volunteer who was also trained in MHFA. Additional re- sources provided to participants during the workshop included a business card which listed key steps on how to help a friend as well as a list of local mental health, as well as a boot bag which contained lollies and a drink bottle with AOTG messaging.		
Fuller et al. (2014)	SPORTSMART, intervention aimed at improving STI screening.<br><br>Two interventions to explore the acceptability and feasibility of football clubs as settings for STI screening (specifically, Chlamydia trachomatis and Neisseria gonorrhoeae) and the potential role of team captains in increasing uptake of screening in young men.<br><br>Pilot cluster-RCT<br><br>The trial coordinator emailed the club contacts prior to the match with brief details of the screening event. On the day, the trial coordinator put up posters in all participating clubs and set-up the test kit collection boxes in the club changing rooms just prior to players' arrival. The interventions were delivered according to randomisation during the usual pre-match or post- match team briefing. Interventions were as follows:<br>1. Captain and poster screening promotion: the team captain delivered a standardised brief screening promotion talk of <5 min duration (figure 1) and then handed each player a test kit and answered any questions from participants.<br>2. Health adviser and poster screening promotion: a sexual health adviser from the study clinic delivered the standardised brief screening promotion talk of <5 min duration and then handed each player a test kit and answered any questions from participants.<br>3. Poster-only screening promotion (comparator arm): posters were displayed that the men were free to read with kits readily available but there was no verbal information given.<br>Men who wished to participate completed a sample kit according to the instructions provided and placed their completed kits back into the secure collection box. Alternatively, men could take the kit away with them for	No adjustments reported.	Apitalising on the non-traditional setting of sport club for men's health interventions.

	later completion and post it back to the clinic in a discreet postage-paid package. All clinical follow-up, including provision of test results via text (SMS) message, was done by clinic staff according to routine standards of care.		
Roncarolo et al. (2015)	Healthy sports stadia program, aimed at supporting health improvement and reduce health inequalities through interaction with local communities and with the people who use the stadia as a place to practice sport, for entertainment or work.<br><br>Methods and process not reported	No adjustments reported	The main assumption of the HS program was that sports stadia can support health improvement and reduce health inequalities through interaction with local communities and with the people who use the stadia as a place to practice sport, for entertainment or work.<br><br>The influence of settings is central in health promotion, since health is determined not only by individual life- styles and by health services, but also by economic, social, environmental, and cultural factors (Dooris, 2012).
Mansfield et al. (2018)	Health and Sport Engagement (HASE) project to engage previously inactive people in sustained sporting activity<br><br><u>1×30 min a week:</u><br>Active participation continues for at least 12 months (1x30mins a week)<br>Community sport interventions continue beyond 12 months as decided by provider<br><br>Examine the associated health and wellbeing outcomes of doing so, and produce information of value to those commissioning public health programmes that could potentially include sport.<br><br>Mixed methods study	No adjustments reported.	Community centered interventions:<br>Locally based initiatives and the role of complex community interventions for public health outcomes.<br><br>Empowerment theory:<br>Sport coaches have a vital role to play in changing social norms around sports through individual and com- munity engagement and empowering or enabling participants to take part in physical activity. Empowerment theory provides a useful theoretical approach for understanding the complexities of raising physical activity levels through community sport. At the community level, empowerment theory investigates people's capacity to influence organisations and institutions which impact on their lives. The theory ad- dresses the processes by which personal and social fac- tors of life enable and constrain behaviours, and this provides the theoretical basis of this study.
Vella et al. (2019)	Four intervention components. Two programs were developed for adolescent males. The first program, called Help Out a Mate, is a 45-min mental health literacy program delivered via a face-to-face workshop within the sports club. The second component, called Your Path to Success in Sport, was designed as an online program that aimed to equip adolescent sport participants with the psychosocial skills that they need to overcome adversities. This	(Adjustments)<br>For the online resilience program, changes were made to address the low numbers of adolescents who registered, and subsequently completed online tasks. The face-to- face workshop content was tailored so that participants gained access to the website/app and completed Module 1 online in the group setting, when possible. Following coach feedback, a	Socioecological model (Eime, Young, Harvey, Charity, & Payne, 2013b) and the settings-based approach to health promotion in sport (Kokko, 2014) AOTG was designed as a multi-component program targeting distinct levels of influence regarding adolescent mental health through sport.<br><br>Coach education program: self-determination

	component incorporated six key modules based on the resilience in sport literature (Fletcher & Sarkar, 2012, 2013; Sarkar & Fletcher, 2014a, 2014b). Each module took 10–15 min to complete.<br><br>x2 60 min face to face workshop with parents and coaches<br><br>Club-based promotional campaign based on principles of social marketing (Stead, Hastings, & McDermott, 2007) was developed to reinforce the key messages of the AOTG program components.	shortened version of the coach program was also developed. This program represented a streamlined version of the full program whereby content was delivered in half the number of workshops/online modules.<br>Smaller number of club engagement officers were employed on a full-time basis. Club engagement officers were also required to be largely responsible for delivering the programs within their designated clubs where previously the research team had delivered most of the programs. To reduce the high (and unsustainable) number of hours that club engagement officers spent in each club, we removed the require- ment for them to be immersed within the clubs. To compensate for this reduced contact, in selecting the designated club champion we required a person of influence within the club (such as a President or Secretary) to enable a stronger influence, easier relationship building, and greater trust. These changes attempted to combine the benefits of both top- down and bottom-up facilitators by streamlining and better integrating program implementation and ownership across stakeholders.	theory: Aim to teach coaches strategies to support their players basic psychological needs.
Hunt et al. (2014)	Football Fans in Training (FFIT)<br><br>12-session, weight management and physical activity group programme with subsequent minimal-contact weight loss maintenance support delivered free of charge.<br><br>Utilises gender-sensitised context, content and style through its delivery at professional football stadia by club community coaches who encourage participative learning, a practical focus through shared experiences of progression towards behaviour change and a light-hearted atmosphere (‘banter’) which supports men in discussing potentially difficult issues.<br><br>In addition to advice on diet, alcohol, and sustainable behaviour change strategies, FFIT focuses on physical activity through an incremental pedometer-based walking programme to encourage greater activity in daily life and pitch-side sessions led by club coaches. FFIT’s location within professional football clubs reflects growing recognition of the potential of sporting organisations to	No adjustments reported.	FFIT exploits the traditionally male environment of football clubs, existing loyalty to football teams and the opportunity to participate in men-only groups to maximise men’s engagement.

	deliver health initiatives to men. Club community coaches receive two days training to ensure that key elements of FFIT are delivered.		
Rookhuijzen & DeVet (2021)	Eight products were added to the range of foods and drinks in two football canteens in the Netherlands for 3 and 15 weeks, serving as a baseline period. In the intervention period, these products were promoted with the use of salience, scarcity, availability and default nudges, for 26 and 16 weeks, respectively. Aside from the collection of sales and revenue data, reach, acceptability, adherence and applicability were measured using observations, questionnaires and interviews.<br><br>The baseline phase lasted 3 weeks in canteen A and 15 weeks in canteen B. This phase was followed by a 26-week and 16-week nudge intervention in canteens A and B, respectively.	(Adjustments reported)<br>Unavailability of some products, and low shelf life, expensive cost, and low sales of some products (e.g., yoghurt and savoury snacks) were removed.<br><br>Products were not aways available - the high number of frequently changing volunteers working in the canteen was put forward as a reason for non-adherence.<br><br>Personnel deemed it too time consuming at busy times. Moreover, after some time, visitors became familiar with the nudge, leading to personnel not willing to enforce the nudge.	Nudge intervention:<br>Using "nudging" - the automatic nature of our dietary decisions to our advantage. Nudging is a strategy that exploits the manner in which automatic processes guide our behaviour to predictably affect it by changing the architecture in which we make decisions, without limiting the set of options. For example, placing unhealthy items out of direct reach reduces the number of participants consuming those items with 53 %.<br><br>Sports context:<br>Football (soccer) is one of the biggest sports of the Netherlands with approximately 3000 clubs with 1·2 million members of all ages and backgrounds. The can- teens that are part of most clubs provide an important source of income. Football clubs, therefore, provide an important setting for the promotion of healthy eating, potentially reaching many people. Moreover, sport facilities can be considered a more manageable and easier to regulate context than locations such as grocery stores for the implementation of nudges, since the choice architect himself/herself is often present on the floor.
Naylor et al. (2015)	Capacity-building intervention (Healthy Food and Beverage Sales; HFBS) on organizational capacity for providing healthy food environments, health of vending machine products, and food policy development in recreation and sport facilities in British Columbia, Canada.<br><br>Twenty-one HFBS communities received training, resources, and technical support to improve their food environment over 8 months in 2009–2010, whereas 23 comparison communities did not.<br><br>Communities self-reported organizational capacity, food policies, and audited vending machine products at baseline and follow-up. Repeated-measures analysis of variance evaluated intervention impact.	Difficulties sourcing food cited.	Publicly funded recreation and sports facilities provide access to a variety of affordable physical activities and are a gathering place for community members for meetings and events. Their substantive contact with children and families makes these facilities an ideal venue for promotion of health and wellness among children.

Gray et al. (2013)	Football Fans in Training (FFIT) programme, which aims to help overweight men (many of them football supporters) lose weight through becoming more active and adopting healthier eating habits.<br><br>(See Hunt et al. 2014)	No adaptations reported	Potential of professional sports organisations to attract men to participate in a range of health promotion initiatives<br><br>Behavioural change techniques associated with a number of theoretical approaches
Dunn et al. (2004)	Personal-Disclosure Mutual-Sharing (PDMS) team building activity delivered at a national championship tournament where the team was required to play three games in a four-day period.<br><br>Included sharing of personal stories and their passion for sport.	No adaptations reported	Shared-cognition:<br>Development of "mutual understanding" among team members is a cornerstone of the team building process. Effective team building interventions targeting either or both task and social cohesion should have a positive influence on performance.
Robertson et al. (2013)	Premier League Health (PLH), a three-year programme of men's health promotion located in 16 top-flight English football clubs targeted men aged between 18 and 35 years from socio-economically deprived communities.	(Adaptations)<br>Interventions varied between clubs, generally reflecting local community needs, and included: educational activities on match days for supporters; weekly physical activity/lifestyle classes; and varied outreach approaches targeted at specific groups of men in local communities. Some interventions were based around match days; some based around the club ground; and others were community-based but club branded and linked. The clubs worked with local community partners in developing the intervention and these partners often contributed time and other resources to support the work. These interventions there- fore were not standardised across the programme, though they were delivered by project staff who received shared education and training in health and behavioural change activities and in working with men on health issues. These project staff were a mix of accredited health trainers, coaching staff and allied health professionals; some of whom already worked within the clubs' community programmes and others who were brought in especially to deliver on the PLH work.	Football club settings and its potential to engage men from socio-economically deprived backgrounds who are not meeting current health lifestyle guidelines, and engaging men with current MH issues<br><br>Particular attention to what generated 'success' and to the role of gender (masculinities) within these processes. The paper situates itself in a gender relations framework understanding masculinities as varying 'configurations of practice' that men move within and between in differing social contexts rather than as more static character traits (Connell 1995, Robertson 2007).
Lauwerier et al. (2020)	CATCH– Community sports for AT-risk youth: innovative strategies for promoting personal development, health, and social cohesion<br><br>delivered over several months (between March and December 2018).<br><br>Sport for development (SFD) training that targeted coaches	No adaptations reported	Sport-for-development (SFD):<br>Provide a setting through which health promotion and prevention messages can more easily reach and be adopted by hard-to-reach populations. SFD initiatives use sports as a vehicle to tackle other issues, such as education, employment, com-munity involvement, health promotion, and prevention.

	to improve their knowledge and skills for the transfer of health promotion messages to at-risk youth. The training program aimed to: (a) increase the awareness and knowledge of coaches on the effects of health behaviour on overall health, well-being, and sport performance (e.g., smoking, physical inactivity, poor dietary habits); (b) increase their awareness and knowledge on the mechanisms to promote the health of CYP; and (c) introduce tools and skills to encourage CYP to participate in community sport activities and adopt a healthier lifestyle.<br><br>Covered topics such as health promotion, healthy living, positive coaching, communication, team dynamics, and conflict. Several strategies were also adopted, including group sessions moderated by one or two tutors with game-based activities, theory and information provision, reflection and discussion exercises, and peer observations. In addition, several individual sessions were planned between and after group sessions. After their first series of four group sessions, each SFD coach had two individual sessions with a job coach, with whom they were already acquainted and had regular encounters regarding their personal (work) trajectories.<br><br>At the end of the program, the job coach planned to have at least one follow-up session to discuss the progress of the coaches towards their own healthy living, as well as their concerns or problems in applying skills to promote health among the youth attending the community sports activities.		Role model (role of the coach):<br>SFD coaches play a vital role in the successes of delivering the activities. Because coaches have regular contact with CYP, they have unique opportunities to build trusted relationships that will enable them to facilitate positive changes in the behaviour and attitudes of CYP<br><br>**Perceived mechanisms of impact:**<br>Creating a safe and trustworthy environment.<br>Building involvement and engagement.<br>Personal health monitoring.<br>Fostering discussion and reflection
Hägglund et al. (2021)	Mindfulness self-reflection intervention (delivered via SMS) to support sustainable high-performance coaching.	(Adjustments)<br>Switched from daily SMS to weekly in Phase 2; switched back to daily SMS in Phase 3 (with new sample from a different sport)	Mindfulness interventions among coaches - coaches who are able to be present and aware are able to better assist athletes and prevent burn out
Hunt et al. (2020)	See Hunt et al. (2014), Kwasnicka et al. (2021), Gray et al. (2013).	(Adaptation of FFIT; Hunt et al., 2020; Hunt et al., 2014; Gray et al., 2013)<br>includes many of the core features of the FFIT program, including self-monitoring of weight and steps, promotion of increased PA and diet modification, setting goals and action planning (Gray et al., 2013).	See Hunt et al. (2014), Kwasnicka et al. (2021), Gray et al. (2013).
Kwasnicka et al. (2022)	Football Fans in Training (FFIT)<br><br>12 weekly group-based 90-min sessions, delivered free of charge to participants by community coaches within	(Adaptation of FFIT; Hunt et al., 2020; Hunt et al., 2014; Gray et al., 2013)<br>Includes many of the core features of the FFIT program, including self-monitoring of weight and	Interventions that are theory-based, designed to align with men's values and interests, group-based, and include multiple behaviour change strategies, are shown to be more effective in changing men's

	professional football clubs.<br><br>Six AFL coaches (4 male, 2 female) were recruited to deliver the program.<br><br>RCT	steps, promotion of increased PA and diet modification, setting goals and action planning (Gray et al., 2013). Aussie-FIT was customised to the AFL context. Additional content was added to coach training and program sessions to support the development of self-regulated behavioural control explicitly by including motivational principles from Self-Determination Theory (SDT) (Ntoumanis et al., 2018).	lifestyle behaviours (Hunt et al., 2020; Sharp et al., 2020).<br><br>Self-determination theory<br><br>**Mechanisms of behavioural change:**<br>Internalisation of motivation
Eather et al. (2020)	MASTER: 15-week coach development intervention on “positive coaching”, which is promoted and fostered through games-based coaching practices and targets six essential elements of sports coaching shown to improve physical, and health and well-being out- comes in children.<br>Following baseline assessments MASTER was implemented using a coach learning process involving three consecutive phases (commencing April 2017): “(i) The MASTER Coach Development Workshop, (ii) Mentoring, and (iii) the Coach Assessment and Reflection“.<br><br>**2-h theory-based face-to-face education workshop**<br>workshop consisted of lecture, discussion and group work activities to introduce the MASTER framework, theoretical underpinnings and practical applications of the MASTER elements, and provided coaches with opportunities to plan sessions based on the MASTER framework (within the existing football curriculum). Coaches were provided with a PowerPoint presentation, printed course booklet, MASTER checklist (Appendix A), MASTER training session notepad, access to video footage examples of the MASTER framework in action, printed (and explained) examples of football training activities and homework tasks.<br><br>**Mentoring**<br>3-week phase involved coaches implementing MASTER elements in their normal training sessions (2 x 1 1⁄2 hour sessions per week) under the guidance of the coach mentor (a member of the research team (BJ)).<br><br>**Coach assessment and reflection**<br>mentor prepared and implement a 2-h training session designed to highlight important aspects of the MASTER framework and games-based coaching practices.	No adaptations reported	Games-based (also known as game-centred) coaching interventions have also grown in popularity in recent years (Harvey & Jarrett, 2014).

Chen (2020)	The Transformational Coaching Workshop is a four-hour behaviour change intervention designed to educate coaches on transformational coaching behaviours (i.e., the four Is) and the effects that these behaviours can have on athlete outcomes.<br><br>Mixed methods design<br><br>17 participants consisted of 10 female and 7 male coaches between the ages of 18 to 68 (Mage = 41.63, SD = 17.24) with one coach not reporting their age.<br><br>Workshop 1:<br>Primarily educated on the concepts of quality participation and transformational coaching. The coaches were also given the opportunity to reflect on their own sport experiences and leadership skills through different interactive activities. The primary activity was a creative non-fiction, narrative story about a new coach of AWAD shadowing a mentor transformational coach during a swimming practice. activities were designed to provide the coaches with opportunities to practice and plan for using transformational coaching behaviours in real- world settings. provided with a workbook resource that contained the workshop activities and the workshop material in greater detail. Additional components of the workshop included video examples, supplementary take-home activities intended for the coaches' learning, and a take-home copy of the Blueprint for Building Quality Participation by the Canadian Disability Participation Project.	(Duration)<br>The duration of Workshop 1 was 3h32m while the duration of Workshop 2 was 3h45m. While the workshops were slightly shorter than the planned four hours, the workshop fidelity checklists (Appendix H) show that most of the workshop components and activities were completed.<br><br>(Deviation)<br>The only exceptions were in Workshop 1, where there was a lack of debriefing after sections of the narrative activity and the facilitator did not provide a story as an example for idealized influence. Additionally, the "how of coaching" activity was also not completed in either workshop but was mentioned to the participants as a take-home activity.	The workshop was a four-hour long group session guided by the full-range leadership model (Bass & Riggio, 2006) and behaviour change theories (Allan, Vierimaa, et al., 2018; Michie et al., 2011).<br><br>Focus on interpersonal behaviours are particularly valuable for CAWAD, as Allan and colleagues (2019) emphasized that training CAWAD on improving their interpersonal skills would be more beneficial than programs that focus on professional knowledge or disabilities awareness.<br><br>Autonomy-supportive coaching intervention: coaches can learn interpersonal behaviours that can improve athletes' quality experiences (e.g., engagement) through formal opportunities<br><br>Designed in behaviour change theories and grounded in the literature of TFL theory (Turnnidge & Côté, 2017).
McGregor (2019)	Psychosocial intervention targeting emotional development in youth academy football.<br>season-long intervention programme with early adolescents in the youth development phase, their coaches, and multidisciplinary support staff.<br><br>A series of four workshops for the youth development phase coaches and support staff. Each workshop was set between three and four weeks apart due to the occasional impact of schedule changes and took place on the academy campus. Three out of the four workshops were delivered in an academy classroom and the final one, in an alternative meeting room outside of the academy building. each of the four workshops were adolescent development and the	No adjustments reported.	Community based and sport specific approach.<br><br>Emotion regulation:<br>During adolescence, changes in two neurobiological systems occur: the socioemotional system and the cognitive control system (Steinberg, 2010). The dual systems model has been used to help explain these changes, with the model identifying the temporal lag that occurs between the structural maturation of the cognitive control and the socioemotional systems (Steinberg, 2008).<br>This intervention targets the emotional regulation system based on the dual systems model.

	impact of emotions, emotion generation and regulation, emotion regulation strategies and support, and positive emotional development through emotion regulation strategies. The sessions began with an interactive activity that linked to the content of the previous session.<br><br>2: Phase two of the intervention involved direct work with the players and was guided by Côte's (1999) Developmental Model of Sport Participation (DMSP). split between the team workshop, small group, and individual player one-to-one sessions.<br><br>Three workshop sessions were delivered at the start of the intervention, with one delivered at the end. the end. Each workshop lasted 45-minutes and took place in an academy classroom (see Appendix I). The discussions in the workshops were geared to promoting mutual sharing between the players. Focus groups to further facilitate engagement. 1 to 1 sessions.		<u>Working mechanisms reported:</u><br>- Awareness [emotional] (personal/others)<br>- Knowledge<br>- Improved understanding of topic area<br>- Improvements generated
O'Brien et al. (2021)	IPEP, a tailored injury prevention program in soccer teams	(Adjustments)<br>Three quarters of IPEP sessions (76%) were completed as originally planned, 11% were not completed as originally planned, and 13% were not completed at all.<br><br>Scheduling changes:<br>by team staff related to other training priorities (e.g., technical and tactical soccer drills, strength and conditioning, cognitive training) or appointments (e.g., video analysis) replacing the originally planned IPEP session.<br>- By club officials, related to scheduling of team training sessions during national team breaks, and the scheduling of friendly games. Managing player workload consisted of staff members cancelling IPEP sessions in view of the total mental and physical workload imposed on players from training, games, and other commitments.	Tailored injury prevention programs engage players, coaches, medical staff, and club administrators as they are developed, increasing their fit to the specific context and reducing the risk of implementation failure
Boelsen-Robinson et al. (2021)	Sugar-sweetened beverage (SSB) reduction initiative<br>- reduce the availability of SSB across all YMCA aquatic and recreation centres.<br><br>Managers and staff at each centre were responsible for introducing and maintaining new drink options and fridge	No adjustments reported.	Nudge:<br>Organisational-level policies have been popular in settings frequented by children and/or that have underlying interests in health promotion. Health-promoting settings such as health services, parks, local councils and sports, recreation and aquatic

	displays.<br><br>Nudge intervention		centres are optimal places to enact policies aimed at encouraging healthy eating, as they are already engaged in other health-related activities, have population reach and may have influence over food retailers in their purview. A common strategy employed in the pursuit of healthier food environments is addressing the availability of healthy and unhealthy options by changing the range of products (i.e., the number of different varieties) and/or the number of units visible to customers (i.e., the number of slots filled in a vending machine).
Sandgren et al. (2022)	Novel Motivational and Psycho-Educational Self-Help Programme for Athletes with Mild Eating Disorder Symptoms (MOPED-A)<br><br>Received a paper-based manual (A4 booklet) of the self-led 'Motivational and Psycho-Educational Self- Help Programme for Athletes with Mild Eating Disorder Symptoms'.<br><br>Athletes were recommended to engage with MOPED-A for 1–1.5 h per week for six consecutive weeks. This included reading one to two chapters a week, completing the relevant activities in each chapter, writing a summary for each chapter, and allowing some time to recap materials (nine chapters in total).<br><br>10-weeks, intervention-arm only, mixed methods.	No adjustments reported.	Increasing motivation:<br>many people with eating disorder symptoms are low on motivation/readiness to change (Vandereycken, 2006), hence a key priority for interventions should be around helping to motivate individuals to initiate and maintain changes to their eating behaviour (Price-Evans & Treasure, 2011). Accordingly, targeting motivation in early interventions for addressing eating disorder symptoms is likely to be important for initiating change in participants (Brewin et al., 2016).<br><br>Self-help interventions are more accessible.<br><br>Intervention Mapping for Adaptation (IMA), a theory-driven, evidence-based and participant-centred protocol for systematically adapting health interventions (Bartholomew et al., 2016).
Petrella et al. (2022)	Supportive care service designed to engage testicular cancer survivors in supportive care by leveraging a community-based sport and exercise model aimed at supporting long-term survivorship outcomes in men living with and beyond testicular cancer.<br><br>Mixed methods study, once per week, 2-h session - lasting 5-weeks. Provided gym membership for single academic term (4-months).	No adjustments reported.	In an effort to engage men in health promotion interventions, sport-based intervention models have been successfully developed and delivered in middle aged men, young men and prostate cancer survivors. These novel interventions engage men in multiple group-based intervention components targeting physical health (e.g., sport play, sideline drills, diet and nutritional support) and mental health (e.g., facilitated social support, psychoeducation on stress reduction and mental health).
Waters et al. (2022)	Assess step consisting of a computer-based assessment program, completed individual at home, in own time (NRL).	In Pilot Phase II with rugby league players, the Assess step was completed in their homes. All Connect step ser- vices were delivered by registered	Ecological systems theory, community- based participatory research frameworks and implementation science frameworks. It recognises

	Reflect:<br>Assessment step generates individual reflect reports that present information regarding mental health symptoms, heat maps, which allows psychologists to follow up with them. Cohort reports also generated for psychologists to work with young person.<br><br>Connect: Dissemination of digital resources, evidence informed modules designed to be flexible and capable of co-delivery 5 sessions, 30-40 mins.	clinical psychologists from the research part of the implementation team (first and eighth authors) as the youth sports program did not have suitably qualified personnel. Connect step services involved the dissemination of digital resources (Life-Fit-Learning Tip Sheets) covering all areas in the Balance Model to parents, coaches and players, and 5 × 30–40 min sessions delivered with players by the registered clinical psychologists (first and eighth authors) including subcomponents from the Healthy Minds, Healthy Bodies, Stay Connected and Strong Minds modules based on results from the Cohort Reflect Reports (see Fig. 1). At the individual level, the registered clinical psychologists (first and eighth authors) received and reviewed the Individual Reflect Reports and followed-up with parents and players to connect them to care via referrals in their local community (see 6 for player mental health and wellbeing outcomes). Cohort Reports only were provided to the manager of the youth sports program so they could integrate the information during program planning.	the interplay of multiple community contexts that influence, and are influenced by, young people, that any effort to reach young people in the communities in which they live must be done in close partnership with community stakeholders and consumers, and that the scientific study of the process and methods of integrating research findings and evidence-based practices within settings is essential to improve the quality and effectiveness of health services and care.
Panza et al. (2022)	Team Talk intervention–a brief workshop-based intervention delivered in a novel sports context to increase adolescent athletes' mental health literacy while linking such messages to group norms and identities.<br><br>Workshop focused on four main components in the following order: (a) orientation and social identity, (b) mental skills training, (c) mental health literacy, and (d) identity and team norms discussion.<br><br>The intervention was delivered by two facilitators. Both facilitators attended mental health first aid certification training prior to delivering workshops. Workshop sessions took place at sites that were convenient for each respective club, including available classrooms, conference rooms, and open space at training facilities.<br><br>Prior to initiating the workshop session, athletes filled-out a pre-survey that included: (a) open-ended items prompting athletes to reflect on their team identity, along with (b) survey items regarding social identity strength and self-efficacy (i.e., baseline values). Following these initial	(Adjustments)<br>Several workshops were scheduled during a large multi-day tournament, with teams completing the workshop during their down time (i.e., before or after their daily games). Although this was convenient for parents and athletes, two workshop sessions were constrained regarding the time available because of scheduling challenges with delivering sessions amidst team activities. Other workshops were delivered before or after weekly team practices. Interventions ranged from 40 to 75 minutes–with an average around 56 minutes (SD = 11 mins). Coaches observed nine of the eleven sessions. Although comparable interventions excluded coaches (see [7]) the inclusion of coaches was integrated into the intervention plan early-on because many coaches of private clubs would only accept the intervention if they could also observe. Although this may present barriers, coach inclusion could be a strategy to ensure that intervention messages are sustained within groups.	Prevalence of sport and the sport setting aligns with the value of multi-level prevention approaches that are valued in broader frameworks regarding community-based health promotion, as well as frameworks regarding promoting mental health in communities (i.e., mental health literacy).<br><br>Mental health literacy is the guiding framework for several recent sport-based interventions to promote mental health. A key goal for targeting mental health literacy is to empower the public with knowledge regarding the wellbeing of themselves and others. An approach grounded in mental health literacy acknowledges that people are commonly unaware of available mental health resources, avoid seeking treatment, and may not be aware of how to recognize problems<br><br>Social norms & social identity theory: Group norms and team environments (group processes)<br>Beyond being a peer setting through which athletes prefer to receive interventions, sport teams entail

	questions, one facilitator presented the material while the other facilitator reviewed team identity item responses, with the goal of crystallizing themes from the team's responses and summarizing these to guide team discussion later in the workshop. The workshop session was guided through content presented via PowerPoint slides or printed 36" X 42" posters when electronic presentations were not possible. Athletes were also provided with a worksheet related to mental skills training as well as a resource card with names and contact information for regional and national resources, such as help lines, mental health service providers, and sport-specific organizations (e.g., 'SafeSport'; safesport.org). In addition to content presented by facilitators, discussion was encouraged among team members to elaborate on key concepts. After introducing the most common responses and reading de-identified responses, the workshop facilitator prompted team members to elaborate on their perspectives. Additionally, participants were prompted to identify team norms regarding the behaviours for members to support one another. Examples of responses to the team discussion activity included: (a) identifying team protocol for checking-in on teammates, (b) identifying a 'buddy' pairing for each person on the team or a team leader with that role, and (c) identifying existing supportive team sayings.		numerous small group processes that can be harnessed. Researchers have indeed reported that when youth perceive their team as a positive environment (e.g., high group cohesion), they tend to report outcomes such as confidence and social connectedness. Team processes can also be leveraged in interventions to achieve aims that fall beyond merely strengthening bonds within teams.
Brooke et al. (2022)	First Episode Psychosis (FEP) recovery intervention<br><br>6-weeks sport programme alongside participant's support workers (i.e., community and peer workers) from the service, including peer workers with a lived experience of psychosis.<br><br>Used various sport activities (e.g. basketball, touch rugby) to promote physical activity, maximise social connectivity, and teach life-skills (e.g. motivation, emotional regulation, and goal-setting) that are relevant and transferrable to other contexts (e.g. school, employment, independent living).<br><br>Three facilitators ran every session (two males and one female, ages 28–36 years).<br><br>Offered weekly for two hours in the afternoon at a local sport facility central to the majority of the service's client base. The structure consisted of the following six phases: (1)	No adaptations reported	Sport as an idea platform due to social agents involved.<br><br><u>Motivation:</u><br>Similar to related work on life skills (Hodge et al., 2012), motivation was targeted through the self-determination theory framework, with the goal of enhancing the participants' sense of competence (e.g. goal setting, skill progression), relatedness (e.g. team building activities), and autonomy (e.g. choice of sporting activities; Ryan & Deci, 2017).<br><br>Bandura's (2001) social cognitive theory – efforts to foster self-efficacy

	welcome and ice-breaker activities (2) mental and physical warm-up (3) mental and physical skill learning (4) play/competition (with rules of the game catered to abilities), (5) mental and physical cool down, and (6) informal social time.<br><br>Breaks were built into the session, and young participants were encouraged to take additional breaks when needed, such as watching from the sideline or engaging in alternative activities (e.g., journaling, ring toss).		

Electronic Supplementary file 7. Implementation data of Process Evaluations Reported.

**Study**	**Fidelity (delivering intervention as intended)**	**Recruitment and reach (dose received and**	**Feasibility**	**Acceptability**
DeCelles et al. (2016)	Research assistants conducted observation of the TOC and MTC interventions (n = 29) to monitor fidelity of the curriculum.	Not reported	Qualitative data reported - focus groups and individual interviews.<br><br>Demonstrated that program curriculum offers feasible approach towards VVMC. Most participants recalled intervention content in detail and cited the "coach story" as a motivational component of the intervention.	Qualitative data reported - focus groups and individual interviews<br><br>Although IDIs and FGDs demonstrated acceptability of curriculum components, participant age posed difficulty, both regarding intervention delivery and VMMC uptake. Coaches generally perceived older participants as uninterested in the soccer-based activity and less inclined to listen to key messages. Moreover, older men were reported to lack motivation to undergo VMMC because they believed that HIV testing and VMMC would make little difference at their age. Numerous interviewees also suggested that participants—particularly older participants—were not interested in using PSI transport to go for VMMC as a group. One reason given by participants was that the status of a man who tests positive would be exposed when not proceeding directly into VMMC (confidentiality).
Santos et al. (2019)	Not reported	Not reported	Not reported	Not reported

Maddison et al. (2019)	Data inferred from recruitment and retention<br><br>12-week, twice-weekly 90-min sessions in Auckland (one during the weekend and one during the working week) and a once-weekly 120–150 min session in Dunedin (held early evening during the working week), which were run at the respective rugby clubs by a RUFIT-NZ coach. In Auckland, the weekend session consisted of a 30-min classroom session followed by a 60-min physical activity session, while the session during the working week consisted of a 90-min physical activity session only. In Dunedin, the session comprised 60min of physical activity and 60–90 min for the classroom session. The number of classroom sessions delivered was the same for the two clubs. The content of the classroom sessions was standardized, so that the men participating at both clubs received the same educational material.	Data inferred from recruitment and retention<br><br>Successful recruitment was defined as recruitment of at least 40 participants at each site over 1month, while successful retention was defined as retention of at least 80% of participants who provided baseline mea- sures. These parameters were set to inform recruitment targets and adherence for a larger trial.<br><br>Recruitment was completed within one month and the recruitment target of 90 participants (40 in Auckland and 50 in Dunedin) was exceeded (n = 96). The retention target of 80% was exceeded, with 97% of controls and 82% of intervention recipients who provided baseline data participating in final follow-up.	Feasibility was assessed by recruitment and retention rates, and acceptability of the intervention.<br><br>127 participants were assessed for eligibility, of whom 3 were excluded due to not meeting the inclusion criteria (either they were not overweight, or they were already meeting the NZ physical activity guide- lines). An additional 22 men registered but were not interested in the study once they received further information.	Quantitative data from participant follow-up survey.<br><br>Overall, 100% of the men from the intervention condition that were followed up reported they liked they pro- gram and would recommend it to other men.
Parnell et al. (2015)	Qualitative data reported on key implementation characteristics.<br><br>Actions of the intervention staff (i.e., community practitioners/coaches) who led the activities were central to programme success; they shared a commitment to providing a meaningful, relevant, and enjoyable programme of health improvement activities.<br><br>The desire of key staff to learn and refine the implementation helped to ensure that the interventions met the needs and satisfied participants.	Posters, flyers, existing contacts and club media channels, such as match day programmes, the club website and magazines were used to recruit OA to ET. Advertisements were also placed in the local press and free newspapers [36]. In some clubs, outreach work was undertaken where OA congregated (including local health centres) and where community leaders were willing to help raise aware- ness of the programme [24,30]. ‘Word of mouth’, whereby participants would tell their friends, was considered central to recruitment [30].<br><br>Successful recruitment often involved getting ‘buy in’ from the participants and attracting those who had OA caring	Not reported	Qualitative data reported - both males and females found the intervention to be acceptable.

		responsibilities<br>One of the most powerful ways of connecting with potential participants was through word of mouth; the social advantages being advocated in these exchanges were important:<br><br>Therefore, following awareness raising sessions under- taken at key settings, potential participants were offered the opportunity to attend a game. This was part of a staged approach, beginning with getting to know the club and from there, to attract them to the programme.<br><br>Reach: Qualitative data presented<br>Found that appeal of football club had positive influence on drawing participants to the program		
Newman et al. (2020)	Data inferred from implementation subscales (see Actual delivery in supplementary file 6)	Not reported	Not reported	Not reported
Eather et al. (2021)	Adherence<br>All eight coaches randomised to the intervention group attended the MASTER Coaching workshop, the mentor assessment session and peer evaluations (excellent adherence to face-to-face program components). During the mentoring phase of the trial only six of the eight coaches and the mentor participating contributed to Facebook discussions, with the other two indicating that face-to-face mentoring was preferred.<br><br>Outcomes of athletes:<br>Seventy-five players (12-16yrs) were in attendance and consented to complete baseline questionnaires (conducted at the start of training session #3 at the courts) and eighty-five players (8-16years) were in	Retention:<br>All sixteen coaches were assessed at baseline and follow-up (100% retention). Of the 131 players who provided consent to be included in the study and filmed during coach assessments, 85 were in attendance and completed game-play assessments and 75 (12-16years) completed questionnaires at baseline and follow-up.	Feasibility of the program was examined using measures of: 1) recruitment (i.e., evaluation of the recruitment process, dissemination of information and obtaining informed consent); 2) retention (i.e., how many coaches completed the program and participated in assessments pre and post-intervention); 3) adherence (attendance at workshop and assessment session, engagement in online forum); and 4) satisfaction (i.e., evaluation of the MASTER program components provided by coaches).	Not reported

	attendance and consented to complete baseline game- play assessment (conducted at the start of training session #4 of the netball season, April-May 2018 prior to competition starting).			
Hurley et al. (2018)	Not reported.	Parents were recruited from sport clubs in two matched communities in Australia. Multiple recruitment strategies were used, including advertisements (with permission) on sport clubs' social media and website pages and on a regional sporting body's website. The lead researcher also visited youth sport clubs on training and match days to increase visibility, develop trust, and facilitate recruitment. Interested parents provided their contact details to receive further information about the study (via phone or email).<br><br>In total, 66 parents (Mage= 44.86 ± 5.2 years), comprised of 51 mothers (77%) and 17 fathers, agreed to participate in the study. In the intervention group, 44 parents (34 women, 10 men) participated in one of five workshops respectively.	Inferred from greater increases in mental health literacy compared to a matched control condition.<br><br>Increased recognition in mental health disorders (depression and anxiety), improved knowledge of help-seeking options, and confidence in providing help.	Not reported
Wynters et al. (2021)	Not reported.	Recruitment:<br>Recruited from the broader AOTG research study during their participation in the program through their soccer club during the 2017 season.	Not reported.	Not reported.
Fuller et al. (2014)	Implementers took field notes to describe the circumstances of each intervention (including weather, match outcome, timing of intervention) to assess fidelity of the interventions in practice.	Identified all potentially eligible amateur clubs in appropriate geographical areas from the Amateur Football Combination listings available on the internet<br><br>Quantitative data reported - 153 men participated in the trial and 90 accepted the offer of screening (59%, 95% CI 35% to 79%).<br><br>In total, 5 of the 18 clubs initially identified had invalid contact details. Of the remaining 13 clubs, 5 did not respond and 8 (62%)	Inferred from acceptability and recruitment.	Quantitative data reported - Acceptance rates were broadly comparable across the arms: captain-led: 28/56 (50%); health professional-led: 31/46 (67%); and control: 31/51 (61%). However, rates varied appreciably by club, precluding formal comparison of arms. No infections were identified.

		indicated that they were willing to participate. Six were chosen based on the willingness of a club representative to meet with the study coordinator and fully discuss the study objectives; the remaining two clubs were placed on a reserve list		
Roncarolo et al. (2015)	Not reported	Recruitment:<br>Associate partners consisted of organizations from eight different countries: UK (2), Finland, Greece, Ireland, Italy, Latvia, Poland and Spain.<br><br>Reach:<br>From the beginning of the program, the number of stadia involved in HS activities varied across the four different evaluations, specifically: 42 stadia in the first evaluation, 59 in the second, 16 in the third and 27 in the final evaluation. The type of sports played in stadia varied significant- ly, mainly depending on the country of origin. The type of sport represented the most was foot- ball, but basketball, rugby, volleyball, cricket and multi-sport stadia, along with minor sport stadia such as greyhound racing, also participated in the program.	Inferred from implementation goals reached	Not reported
Mansfield et al. (2018)	Not reported	Increased coach competence in delivering PA interventions	Data inferred from outcome of intervention	Not reported
Vella et al. (2019)	High fidelity: Assessed whether intervention was being delivered as intended by the program developers and in line with the program model (Breitenstein et al., 2010).	In total, 271 adolescents participated in the Help Out a Mate program (95.7%), 251 (88.7%) participated in the Your Path to Success program, and 239 (84.5%) participated in both programs. The majority of Your Path to Success workshop attendees (77%) logged in to the online program, and 47% of those who logged in completed all six modules. Alongside significantly improving the dose delivered and received, the engagement team in Phase III spent only 0.67 h per participant delivering programs, compared to 4.8h in Phase II.	Assessed cost of program	Qualitative data reported - inferred from whether participants were willing to complete full program. Changes to program duration were subsequently made.

Hunt et al. (2014)	Reported elsewhere	Reported elsewhere	Reported elsewhere	Qualitative data reported - inferred from what factors that attract and keep participants in the program
Rookhuijzen & DeVet (2021)	Inferred from adherence:<br><br>Observations made by the researcher during visits of the canteens	Reach - measured by counting the number of people visiting the canteen on a single day, collecting sales figures and data on the amount of transactions made that day and through questionnaires that were filled in on the same day	Measured as applicability: whether the IV can be implemented in similar settings (mixed methods)	Acceptability was measured through questionnaires targeted at visitors and through semi-structured interviews with board members and canteen personnel.<br><br>1-5 likert scale to investigate how acceptable participants found the intervention to be
Naylor et al. (2015)	Not reported	Not reported	Inferred through outcomes	Not reported
Gray et al. (2013)	Not reported	Unable to estimate reach	Reported elsewhere	Inferred from perceptions of program
Dunn et al. (2004)	Not reported	Not reported	Not reported	Not reported: inferred from athletes' positive subjective experiences
Robertson et al. (2013)	Not reported	Recruitment:<br>advertising projects/ interventions on screens at games; local newspaper adverts; recruitment via community groups (e.g. Children's Centres, drug rehabilitation programmes and job centres); and GP referrals.	Inferred from participant interviews, aimed at understanding:<br>(1) 'What are the underlying processes and features at work within the programme that lead to success', and (2) 'Where and how does gender (masculinities) appear in these processes'.	Inferred through qualitative data on program components
Lauwerier et al. (2020)	Inferred from direct observations of intervention delivery.	Inferred from direct observations of intervention delivery.	Assessed with RE-AIM facets: Reach, dose, Fidelity, Acceptability	Inferred from direct observations of intervention delivery.
Hägglund et al. (2021)	Daily SMS-intervention over 5 consecutive competition days during the championship that yielded an 89% response rate, which provided a high enough fidelity and reach	daily SMS-intervention over 5 consecutive competition days during the championship that yielded an 89% response rate, which provided a high enough fidelity and reach	Not reported	Not reported

Hunt et al. (2020)	See Hunt et al. (2014), Kwasnicka et al. (2021), Gray et al. (2013).	See Hunt et al. (2014), Kwasnicka et al. (2021), Gray et al. (2013).	See Hunt et al. (2014), Kwasnicka et al. (2021), Gray et al. (2013).	See Hunt et al. (2014), Kwasnicka et al. (2021), Gray et al. (2013).
Kwasnicka et al. (2022)	Not reported	Reported as uptake	Qualitative data reported:<br>Location of intervention	Qualitative data reported
Eather et al. (2020)	Reported as part of feasibility measure	Reported as part of feasibility measure	Quantitative data reported - using measures of **recruitment** (i.e., evaluation of the recruitment process, **dissemination** of information and obtaining informed consent), **retention** (i.e., how many coaches completed the program and participated in assessments pre and post-intervention), **adherence** (i.e., the degree to which coaches followed the MASTER program), and **satisfaction** (i.e., level of coach satisfaction of the program measured via a short evaluation questionnaire).	Reported as part of feasibility measure
Chen (2020)	Reported with workshop fidelity checklist and field notes	Not reported	Not reported	Not reported
McGregor (2019)	Reported as part of uptake	Reported as uptake of intervention - how the participants engaged with the intervention, their exposure to the content, their views on the various components and activities, and what factors lay within the delivery and participants' experiences.	Not reported	Not reported
O'Brien et al. (2021)	Data reported in process evaluation findings	Not reported	Not reported	Not reported
Boelsen-Robinson et al. (2021)	Not reported	Not reported	Inferred from quantitative data - customer perspectives and checklist audits	Inferred from quantitative data - customer perspectives and checklist audits

Sandgren et al. (2022)	Not reported	Recruitment & retention:<br>Recruitment data were recorded to determine the feasibility of recruiting participants to the study and to determine the recruitment rate (N recruited ÷ recruitment time [weeks]; Walters et al., 2017). Feasible recruitment was determined as the ability to recruit be- tween 24 and 30 participants, which has been suggested to be sufficient for feasibility studies (Julious, 2005; Lancaster et al., 2004; Sim & Lewis, 2012). Retention data were recorded at T2 and T3 to determine the proportion of participants who were still actively participating in the study and retention was considered successful if ≥ 80% of participants were retained at T3 (Thomas et al., 2004). Measures were determined to be acceptable to participants if there was <10% missing data which was missing completely at random (Bennet, 2001), in addition to ≥80% survey completion rates at T2 and T3 (Thomas et al., 2004). Research procedures were determined acceptable if there were no significant burdens or issues raised by participants (Palmcrantz et al., 2015).	Quantitative outcomes reported - following existing feasibility guidelines (Bowen et al., 2009; Orsmond & Cohn, 2015), and T3 questionnaires<br><br>Qualitative outcomes reported - one-to-one interviews	2 parts:<br>- Acceptability of measures and research procedures<br>- Acceptability of intervention<br><br>Quantitative and qualitative feedback suggested the format, delivery, content and dosage of MOPED-A were acceptable.<br><br>"Participant response data indicated a high acceptability of measures; missing data were minimal (5.2%) and Little's MCAR test demonstrated that this was completely at random (p > 0.05) and therefore replaced. "<br><br>Acceptability of intervention: Twenty-nine participants completed the evaluation questionnaire at T2; 15 of these participants (52%; female: n = 14; male: n = 1; Mage = 25.8 years, SD = 8.06, range: 18–46 years) also completed an interview and chose to do this over the phone. Twenty-eight participants completed the follow- up survey at T3.<br><br>IV completion rate and use
Petrella et al. (2022)	Reported in discussion	Not reported	Reported in discussion - included investigation of research procedures and intervention components	Assessed using post intervention survey and focus group data
Waters et al. (2022)	Yes - reported actual delivery	Penetration (degree of integration within each setting): proportion of young people	Feasibility (ease of implementation), practicality and ease of application	Acceptability (stakeholder and consumer satisfaction): extent to

		within each setting who were accessed relative to organisation goals	NRL players, the main obstacle was a higher rate of non-completions, possibly because of completing the Assess step at home rather than in school.	which end-users of the way in which the Life-Fit-Learning system was implemented within each organisation found the experience to be satisfactory and informative.
Panza et al. (2022)	Yes - reported actual delivery	Penetration (degree of integration within each setting): proportion of young people within each setting who were accessed relative to organisation goals	Feasibility (ease of implementation), practicality and ease of application<br><br>NRL players, the main obstacle was a higher rate of non-completions, possibly because of completing the Assess step at home rather than in school.	Acceptability (stakeholder and consumer satisfaction): extent to which end-users of the way in which the Life-Fit-Learning system was implemented within each organisation found the experience to be satisfactory and informative.
Brooke et al. (2022)	Using Bowen et al. (2009) framework	Not reported	Assessed through recruitment statistics, participant records (i.e. attendance, session engagement, and completion of questionnaire records for each participant), a record from the programme facilitators (i.e. exact session activities, reflections/observations, and modifications made), and feedback from young and staff participants. Intervention components were assessed through session engagement, session records, and feedback from young and staff participants.	Using Bowen et al. (2009) framework

Electronic Supplementary file 8. Characteristics of excluded studies in full text.

**Reason for exclusion**	**Studies**
No explicit mention of process evaluations	Brinkley et al. (2022),<br>Kihl et al. (2014)<br>Duff 2007<br>Abotsie 2020<br>Rutherford 2014<br>Hutchesson 2021<br>Caron 2018<br>Owoeye 2020<br>Whitley 2016<br>vanRens 2019<br>Pringle 2021<br>Pringle 2016<br>vanKruijsbergen 2020<br>Wilcock 2021<br>Bowley 2018
Not an intervention (i.e., service provision)	Keegan 2022<br>Keegan 2020
Duplicates	Newman 2021<br>DeCelles 2016<br>DeCelles 2016
Not in sport setting	Gee 2022<br>Carr 2021<br>AElMasri 2021<br>Titze 2018<br>Casey 2011<br>Bruner 2010<br>Bean 2015<br>Iachini 2014<br>Kerr 2018<br>Terry 2014<br>Newland 2013<br>Robbins 2016<br>Welsby 2014<br>Waqa 2013<br>Merrill 2018<br>Breslin 2018
Not original empirical research	Krølner 2012<br>Sewry 2017<br>Ooms 2015<br>Hoekstra 2014<br>Bolton 2018<br>Randall 2019

	Marlier 2015
No full text	Enrique Garcia Bengoechea 2022
Did not perform process evaluation	Patafio 2021<br>Taylor 2021<br>Kellmann 2011<br>Moore 2021<br>Røynesdal 2021<br>Delaunay 2021<br>Jackson 2015<br>Kwasnicka 2020<br>Delva 2010<br>Bunn 2016<br>Anokye 2018<br>Hurley 2020<br>Hurley 2021<br>Maddison 2019<br>Gipson 2018<br>Obling 2019<br>Olmedilla 2019<br>Santhya 2019
